# Coordination‐Locked Engineering to Achieve Narrowband Room Temperature Phosphorescence in Non‐Rare Earth Metal–Organic Frameworks

**DOI:** 10.1002/advs.202516309

**Published:** 2025-10-22

**Authors:** Wenlei Zhang, Jinpeng Li, Mengyao Wang, Yining Zhao, Xi Zhang, Hongfeng Li, Jing Liu, Tianyang Li, Meng Zhang, Fei Chen, Zhongyi Liu, Chao Lu

**Affiliations:** ^1^ College of Chemistry, State Key Laboratory of Coking Coal Resources Green Exploitation Zhengzhou University Zhengzhou 450001 China; ^2^ State Key Laboratory of Chemical Resource Engineering, College of Chemistry Beijing University of Chemical Technology Beijing 100029 China; ^3^ School of Intelligent Manufacturing Huzhou College Huzhou 313000 China

**Keywords:** coordination‐locked phosphorescence amplification, metal–organic frameworks, multilevel information recognition, narrowband phosphorescent, non‐rare‐earth

## Abstract

Non‐rare earth (RE) narrowband phosphorescent metal–organic frameworks (MOFs) remain a great challenge due to spectral broadening from overlapping emissions in complex coordination systems. Here, the first non‐RE narrowband room temperature phosphorescent (RTP) MOF crystal, **Sr–tbc**, constructed via coordination‐locked phosphorescence amplification (**CLPA**) of a tailored phosphor, 1‐(4‐carboxyphenyl)‐1H‐1,2,4‐triazole‐3‐carboxylic acid (H_2_tbc), is presented. **Sr–tbc**, with 1D coordination chains, exhibits a significant narrowband RTP emission over a wide excitation wavelength range of 280–360 nm with a minimum half‐height full width value of 28.5 nm. This exceptional narrowband emission is attributed to the targeted coordination between triazolinic acid and Sr ions. This special coordination structure selectively enhances specific RTP emission and restricts vibrational dissipation through rigid molecular stacking. Furthermore, **Sr–tbc** shows potential in high‐resolution afterglow displays and multilevel information encryption. This work reports the first case of non‐RE narrowband phosphorescent MOF crystals and establishes a novel **CLPA** strategy for efficient RTP materials.

## Graphical abstract

In this contribution, the coordination‐locked phosphorescence amplification (CLPA) strategy is developed to successfully construct the first non‐rare‐earth narrowband RTP MOF crystal, **Sr–tbc**. In addition, **Sr–tbc** shows potential applications in high‐resolution afterglow display and multilevel information recognition.

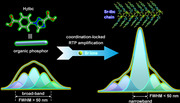

## 1 Introduction

Solid room temperature phosphorescent (RTP) materials with narrowband emissions demonstrate significant promise for optoelectronic applications.^[^ ^1^, ^2^, ^3^ ^]^ These narrowband phosphorescent materials display full width at half maximum (FWHM) values below 50 nm, contributing to remarkable color purity and high quantum efficiency. More interestingly, their transformative potential is particularly evident in ultrahigh‐definition displays, high‐resolution bioimaging, and multiplexed sensing.^[^ ^4^, ^5^, ^6^ ^]^ Consequently, it is urgent to develop efficient narrowband phosphors with minimal spectral broadening to achieve the high color purity for next‐generation lighting and displays. Among these, metal–organic frameworks (MOFs) have recently emerged as a frontier platform for engineering RTP materials, due to their unique combination of ordered porosity, programmable metal–ligand interactions, and rigid chromophore arrangements.^[^ ^7^, ^8^, ^9^, ^10^, ^11^, ^12^ ^]^ Despite these advantages, the existing narrowband phosphorescent MOFs predominantly relied on rare earth (RE) ions (e.g., Eu^3+^‐based systems with f–f transitions yielding FWHM < 10 nm).^[^ ^13^, ^14^ ^]^ These RE‐based RTP systems suffer from inherent limitations, including restricted color tunability, scarcity‐driven costs, and poor environmental stability.^[^ ^15^ ^]^ Moreover, the existing non‐RE broadband MOFs (FWHM = 70–150 nm) fail to meet advanced optoelectronics.^[^ ^16^, ^17^, ^18^, ^19^ ^]^ Therefore, in order to eliminate RE dependency and expand emission, it is essential to design non‐RE narrowband phosphorescent MOFs for scalable high‐color‐purity applications and photonic paradigm shifts.

To address the above challenges, various attempts have been conducted.^[^ ^20^, ^21^, ^22^, ^23^ ^]^ First, the structural rigidification through crystallographic confinement has been used to suppress non‐radiative decay. Yan's group synthesized heterometallic MOFs, achieving a 195.4 ms phosphorescent lifetime and 9.1% quantum yield utilizing coordination solvents to stabilize framework and Sr/Ba atoms to boost spin–orbit coupling (ISC),^[^ ^24^ ^]^ and they achieved efficient fluorescence/phosphorescence hybrid white‐light emission in Zn‐IPA MOFs via pressure‐modulated structural rigidity and coordination configuration, enhancing triplet exciton distribution and boosting photoluminescence quantum yield to 81.3%.^[^ ^25^ ^]^ Second, the optimization of coordination modes and the introduction of heavy atoms have emerged as a powerful tool to boost spin‐orbit coupling (SOC).^[^ ^26^, ^27^, ^28^ ^]^ The heavy‐atom effect operated through synergistically accelerating ISC, suppressing spin‐lattice relaxation, minimizing electron‐phonon coupling, and optimizing radiative transition probabilities.^[^ ^29^, ^30^ ^]^ Notably, these advances enabled high‐performance RTP without depending on rare metals through precision coordination chemistry. Inspired by these findings, we speculate that the spectral broadening bottleneck in non‐RE MOF phosphors could be broken by selectively enhancing single emission channel and suppressing competing emissions via heavy‐metal coordination design.

**Figure 1:**
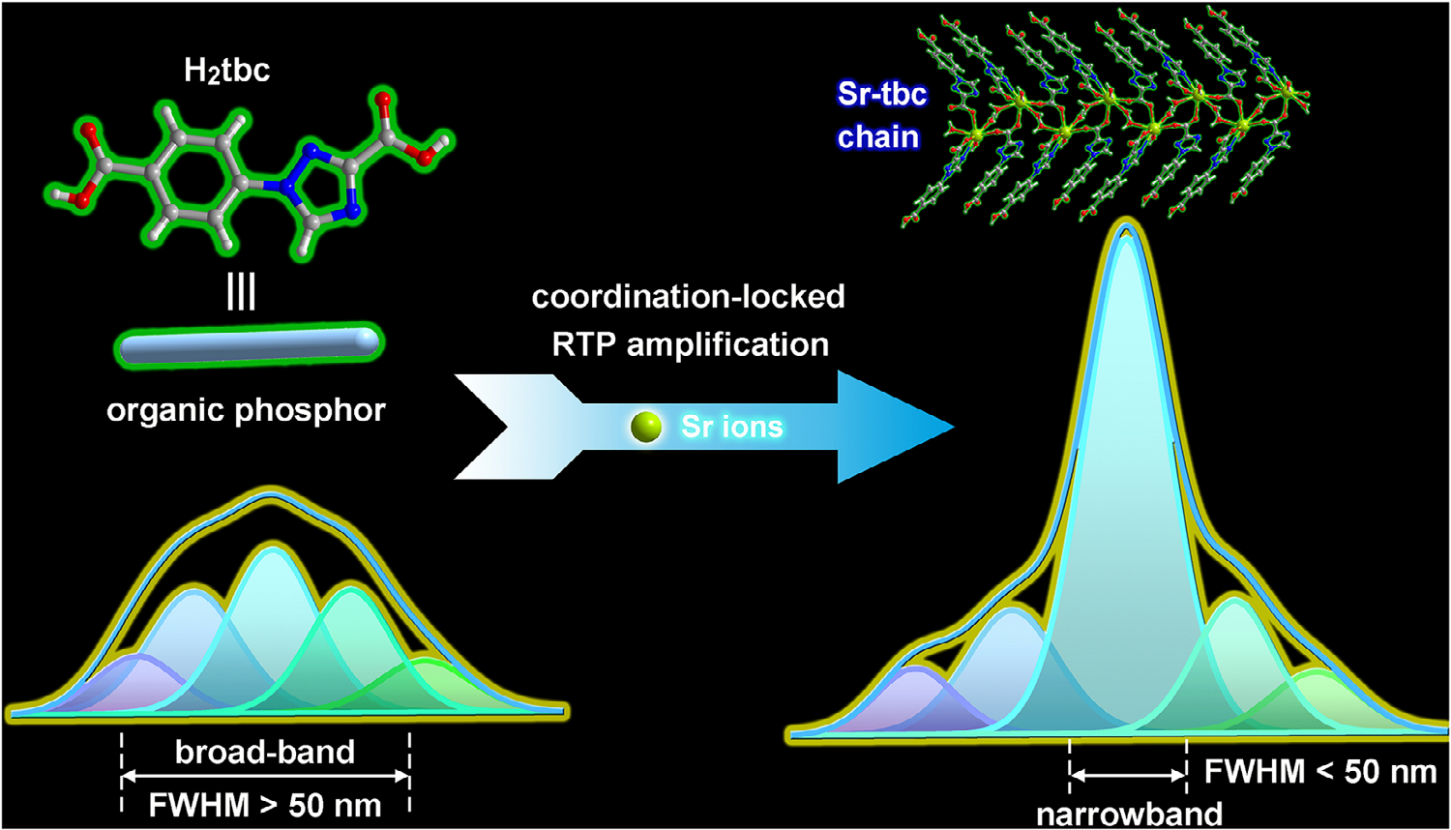
Design diagram of narrowband **Sr–tbc** crystals through coordination‐locked phosphorescence amplification strategy. The 1D **Sr–tbc** crystal coordination chains are constructed by controlling the coordination dimensions of organic phosphors (H_2_tbc), and the 1D **Sr–tbc** displays narrowband RTP emission (FWHM = 28.5 nm) due to the targeted enhancement of specific phosphorescent emission.

Herein, we achieved a narrowband emission of non‐RE MOFs for the first time by engineering the coordination dimension of 1‐(4‐carboxyphenyl)‐1H‐1,2,4‐triazole‐3‐carboxylic acid (H_2_tbc) phosphor with metal ions (**Figure** **1**). Two kinds of MOFs, a novel [Sr(Htbc)_2_(H_2_O)_3_]_n_ crystal (denoted as **Sr–tbc**) featuring parallel 1D Sr─O─C─O─Sr coordination chains and a reported [Cd(tbc)(H_2_O)_2_]_n_ crystal (denoted as **Cd–tbc**) featuring 2D coordination frameworks, were synthesized. In comparison to the 2D **Cd–tbc** frameworks, the 1D **Sr–tbc** MOFs exhibited a typical narrowband emission at 548 nm with the smallest FWHM value of 28.5 nm. This feature was ascribed to the fact that the monodentate coordination selectively boosted the luminescence efficiency of the triazolinic acid moiety. Furthermore, **Sr–tbc** crystal displayed ultra‐long RTP lifetime (up to 552 ms) at an emission wavelength of 500 nm. The excellent RTP performances were attributed to the designable coordination dimension, which enhanced the stacking efficiency of the organic ligands and improved structure rigidity. These structural characterizations could reduce the non‐radiative transitions of triplet excitons and ultimately contribute the narrowband emission and extend the RTP lifetime. This work reports the first case of the narrowband emission from non‐RE MOFs with ultra‐long afterglow, providing a novel strategy for developing high‐color‐quality afterglow.

## 2 Results and Discussion

### 2.1 Structure Characterizations of RTP Materials

**Figure 2:**
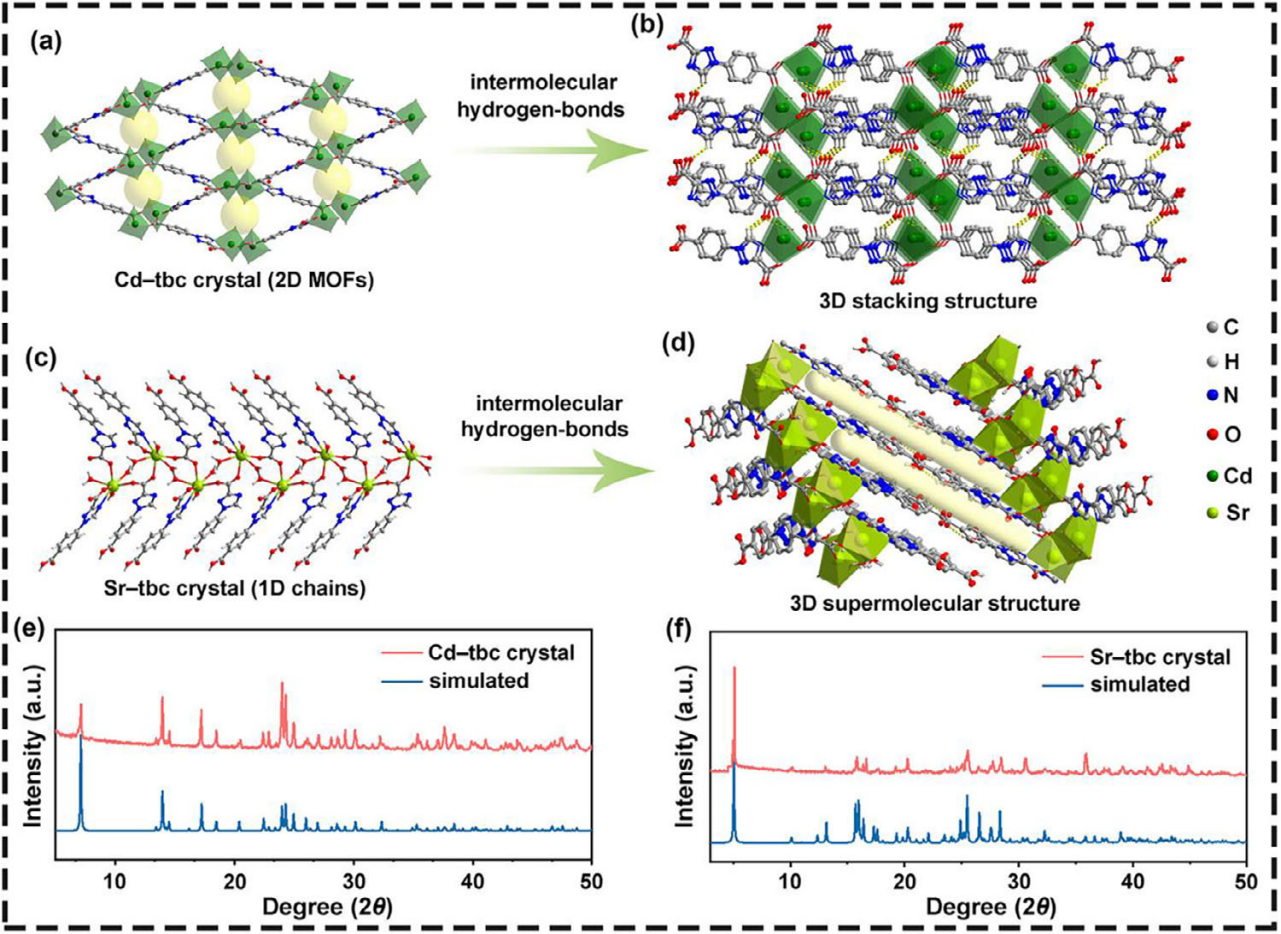
Crystal structures of **Cd–tbc** and **Sr–tbc** samples. The partial structures of a) 2D **Cd–tbc** layer and c) 1D **Sr–tbc** chains; b) The stacking diagrams of 3D supermolecular structures of b) **Cd–tbc** and d) **Sr–tbc** (intermolecular hydrogen bonds are indicated by dotted lines, and some hydrogen atoms have been omitted for a clearer view); The tested and simulated XRD patterns of e) **Cd–tbc** and f) **Sr–tbc** samples.

Two types of tbc‐based MOFs, **Cd–tbc** and **Sr–tbc**, were synthesized through a straightforward solvothermal method (Figure S1, Supporting Information). Their crystal structures were confirmed by single‐crystal X‐ray diffraction (XRD) measurements. The **Cd–tbc** crystal, a typical 2D MOFs with regular pore distribution (**Figure** **2**a; Figures S2 and S3, Supporting Information), spontaneously self‐assembled into a 3D supramolecular structure through intermolecular hydrogen bonds (Figure 2b; Figure S4, Supporting Information).^[^ ^31^ ^]^ In contrast, the **Sr–tbc** crystal, a 3D supramolecular structure with regular pore distribution, was assembled from 1D **Sr–tbc** coordination chains interconnected by intermolecular hydrogen bonds (Figure 2c,d; Figure S5, Supporting Information). Notably, the novel **Sr–tbc** crystal should be categorized as 1D MOFs.^[^ ^32^ ^]^ Thus, the two MOFs displayed distinct coordination dimensions, and their detailed structural data were provided in the supplementary information (Tables S1 and S2, Supporting Information). Furthermore, the phase purity and crystallinity of both **Cd–tbc** and **Sr–tbc** were further analyzed using powder XRD measurements. The experimental XRD patterns of both crystals matched their simulated counterparts, with characteristic reflections for **Cd–tbc** at 7.1° (1 0 0), 14.0° (1 1 −1), 17.2° (2 1 −1), 24.0° (1 2 0), and 24.3° (3 1 0) and for **Sr–tbc** at 5.1° (0 0 2), 15.8° (1 1 0), 20.3° (0 0 8), 25.5° (1 2 4), and 28.5° (0 2 8) (Figure 2e,f). This consistency signified that both the prepared crystalline framework materials owned high crystallinity, making them suitable for the investigation of photophysical properties.

### 2.2 Narrowband RTP Property

**Figure 3:**
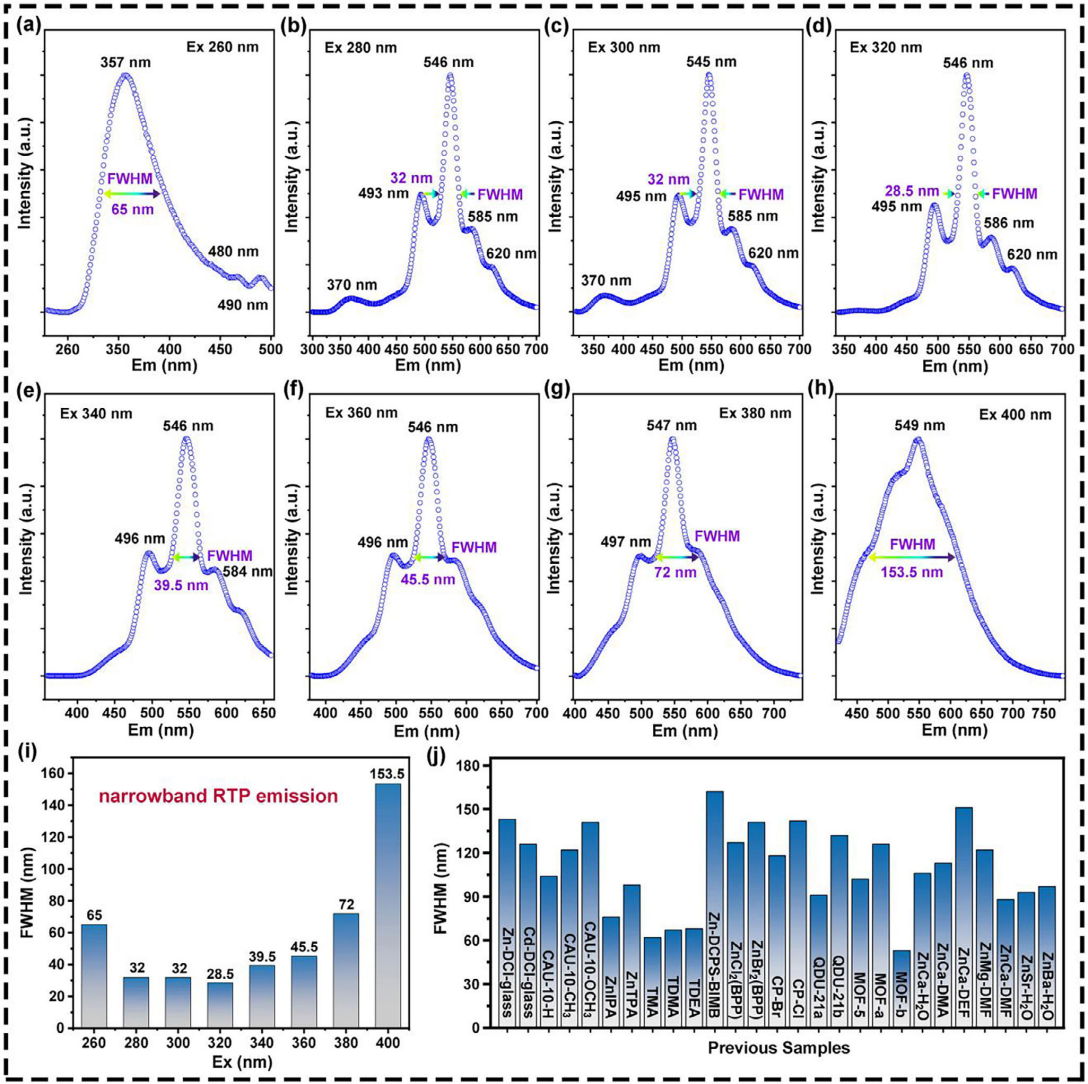
The narrowband RTP emission features of prepared **Sr–tbc** crystal. a–h) Different FWHM values varies with excitation wavelengths in normalized photoluminescence spectra; The comparative FWHM values of i) **Sr–tbc** and j) other reported MOF materials. All tests were conducted at room temperature.

Normalized photoluminescence (PL) spectra were conducted to understand the optical property of the constructed **Sr–tbc** crystals. First, due to the coordination structures, **Sr–tbc** crystals exhibited the distinct multi‐peak emission features with partial overlap (Figure S6, Supporting Information). Notably, **Sr–tbc** crystals displayed the appealing narrowband RTP emission features (FWHM < 50 nm) within a specific range of excitation wavelengths (Ex) spanning from 280 to 360 nm and the corresponding emission wavelength (Em) was ≈546 nm (**Figure** **3**a–h). Specifically, the FWHM values of **Sr–tbc** crystals at various excitation wavelengths were 65 nm (Ex = 260 nm), 32 nm (Ex = 280 nm), 32 nm (Ex = 300 nm), 28.5 nm (Ex = 320 nm), 39.5 nm (Ex = 340 nm), 45.5 nm (Ex = 360 nm), 72 nm (Ex = 380 nm), and 153.5 nm (Ex = 400 nm), respectively (Figure 3i). The **Sr–tbc** crystal exhibited a significantly narrower experimental FWHM of 28.5 nm in comparison to the previously reported MOF phosphors,^[^ ^5^, ^20^, ^24^, ^33^, ^34^, ^35^, ^36^, ^37^, ^38^, ^39^ ^]^ as demonstrated by a comparative analysis of 27 MOF samples compiled from the prior literatures (Figure 3j). This narrowband RTP emission would grant **Sr–tbc** crystals high value in applications, such as high‐resolution imaging, high‐quality display, anti‐counterfeiting encryption, and multi‐channel sensing. Furthermore, when excitation wavelengths range from 260 to 300 nm, the significant ultraviolet RTP emissions (Em < 380 nm) were observed, including the peaks at 357 nm (Ex = 260 nm), 370 nm (Ex = 280 nm or 300 nm) (Figure S7, Supporting Information). This unique ultraviolet RTP emission, characterized by high energy, may endow **Sr–tbc** crystals with the capability for secondary excitation, further enhancing their application potential.^[^ ^40^ ^]^ Therefore, **Sr–tbc** crystals exhibited the attractive narrowband RTP emission and unique ultraviolet RTP emission due to their controlled RTP emission process.

### 2.3 Structure–Property Relationship

**Figure 4:**
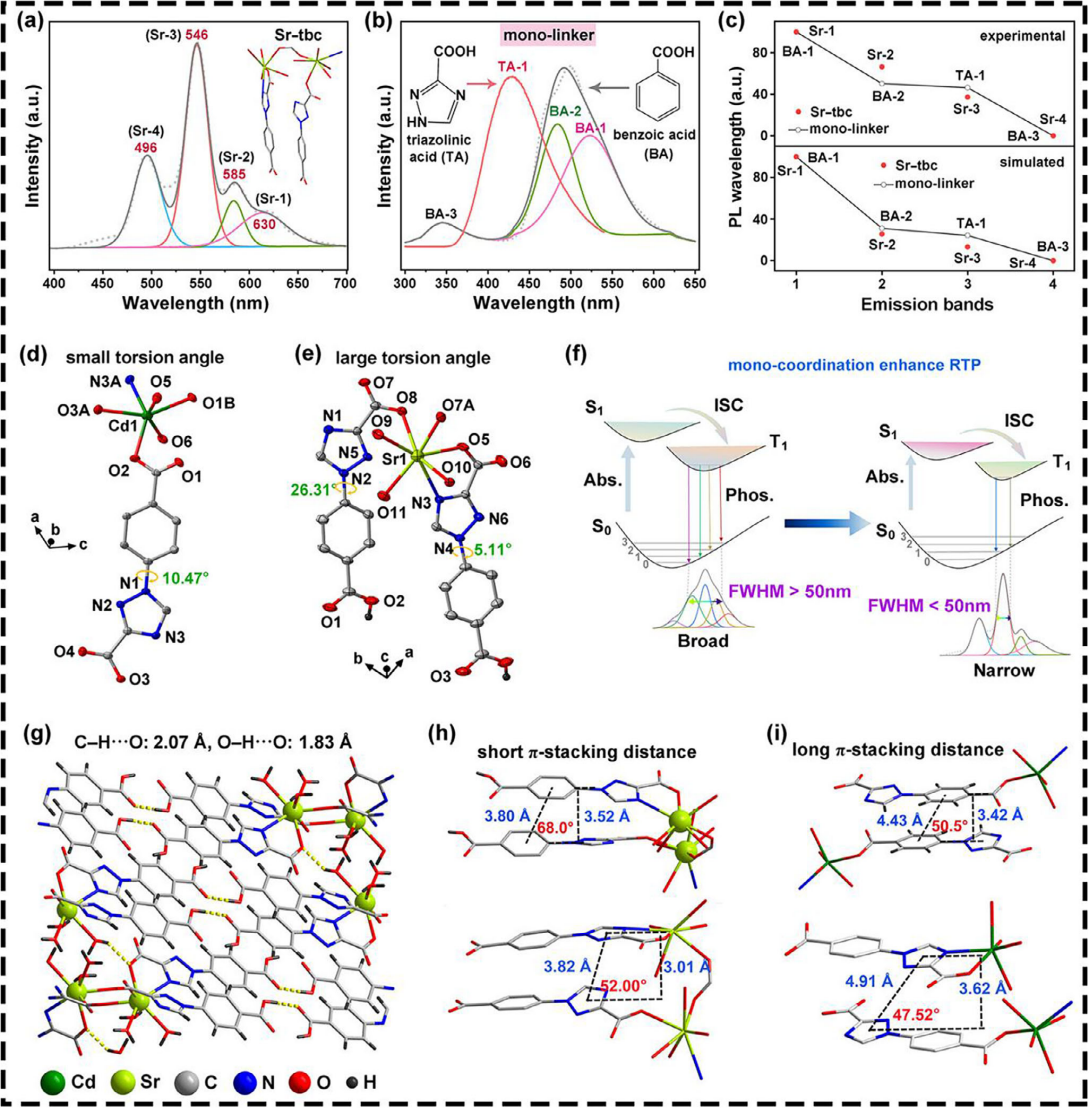
Mechanistic origin of narrowband phosphorescence in **Sr–tbc**. Deconvoluted phosphorescence emission bands of a) **Sr–tbc** (Sr1, Sr2, Sr3, and Sr4), and b) mono‐linkers (benzoic acid: BA‐1, BA‐2, BA‐3; triazolinic acid: TA‐1); c) Correlation analysis between normalized emission wavelengths (0–100) of **Sr–tbc**, benzoic acid, and triazolinic acid, including simulated and tested wavelengths; The asymmetric structural unit of d) **Cd–tbc** and e) **Sr–tbc** (some hydrogen atoms have been omitted for a clearer view); f) The schematic process of narrowband phosphorescence via site‐selective coordination engineering. The stacking diagrams of 3D g) **Sr–tbc** (intermolecular hydrogen bonds are indicated by dotted lines); Intermolecular stacking of tbc dimer in (h) **Sr–tbc** and i) **Cd–tbc** crystals.

Theoretical simulations and spectral deconvolution of RTP emission bands were systematically performed to elucidate the origin of narrowband RTP in **Sr–tbc** crystals. The emission peaks of **Sr–tbc** samples were deconvoluted into the four peaks (Sr1: 630 nm, Sr2: 585 nm, Sr3: 546 nm, and Sr4: 496 nm) (**Figure** **4**a). And those of the free H_2_tbc linker were also deconvoluted into the four peaks (TB1: 629 nm, TB2: 589 nm, TB3: 554 nm, and TB4: 512 nm) (Figure S8, Supporting Information). These results revealed that the narrowband RTP at 546 nm in **Sr–tbc** corresponded to the TB3 emission (554 nm) of the H_2_tbc linker, with a hypsochromic shift attributed to coordination‐induced structural rigidity.^[^ ^41^ ^]^ Further investigations on the mono‐linkers, triazolinic acid (**TA**) and benzoic acid (**BA**), demonstrated the distinct RTP behaviors (Figures S9 and S10, Supporting Information). The BA linker exhibited the three resolved emission bands (BA‐1: 524 nm, BA‐2: 435 nm, BA‐3: 345 nm), while TA showed a single emission peak at 428 nm (TA‐1) (Figure 4b). The experimental emission order (BA‐1 > BA‐2 > TA‐1 > BA‐3) aligned with theoretical predictions (Table S3, Supporting Information), confirming the reliability of computational models. Crucially, the narrowband emission at 546 nm (Sr3) in **Sr–tbc** was conclusively assigned to the **TA** moiety of the H_2_tbc linkers. Strong correlations between simulated and experimental emission wavelengths for both H_2_tbc linker (Sr1, Sr2, Sr3, and Sr4) and mono‐linkers (BA‐1, BA‐2, TA‐1, BA‐3) (Figure 4c) further validated this assignment. Furthermore, both 1H‐1,2,4‐triazole‐1‐benzoic acid (**TBA**) and 1‐(phenyl)‐1H‐1,2,4‐triazole‐3‐carboxylic acid (**BTA**) demonstrated the multi‐peak emissions similar to those of H_2_tbc ligand (Figures S11 and S12, Supporting Information). These similarities further demonstrated the synergistic integration of the benzene ring and the triazole ring. The enhanced RTP intensity and spectral narrowing were attributed to selective coordination between triazolinic acid and Sr^2+^ ions, which suppressed non‐radiative decay pathways and stabilized the Sr3‐dominated emissions (Figure 4f).^[^ ^42^ ^]^

The fine structures of these crystals were analyzed in detail in order to comprehend the significant relationship between the optical properties and crystal structure of these constructed tbc‐based crystals. As observed from the asymmetric structural units of **Cd–tbc** and **Sr–tbc** (Figure 4d,e), it was evident that both tbc^2−^ chromophores of two MOFs exhibited distorted geometries, with torsion angles between the triazole and benzene units ranging from 5.11° to 26.31°. In contrast to the twisting angles of **Cd–tbc** (10.47°), the asymmetric structural unit of **Sr–tbc** displayed the two notably different torsion angles (5.11° and 26.23°), potentially contributing to its multimodal emissions and extended phosphorescence lifetime.^[^ ^43^ ^]^ Essentially, these distorted conformations could effectively prevent phosphorescence from self‐quenching from heavy aggregation.^[^ ^44^ ^]^ Additionally, the torsional conformation could aid in the spatial separation of the highest occupied molecular orbital (HOMO) and the lowest unoccupied molecular orbital (LUMO), which was conducive to reducing the singlet‐triplet energy gap (ΔE_ST_).^[^ ^43^ ^]^ This reduction further promoted the generation of triplet excitons by enhancing the ISC process, playing a pivotal role in phosphorescence production.

The molecular conformation of **Cd–tbc** and **Sr–tbc** metal–organic hybrids was solidified through intermolecular hydrogen bonds and coordination bonds between the metal ions and the ligands (Figure 4g; Figure S13, Supporting Information). Specifically, these interactions could stabilize the conformation of **tbc** ^2−^or **Htbc** ^−^ by dampening the vibration of the carboxyl group and hindering the rotation of the aromatic ring, which could significantly enhance the phosphorescence properties of the **Cd–tbc** and **Sr–tbc** hybrids.^[^ ^41^ ^]^ In the case of **Cd–tbc** and **Sr–tbc** hybrids with the different coordination dimensions, the molecular stacking regulated by coordination interactions was explored. In **Cd–tbc** crystals, the bond lengths of C─H─O and O─H─O were 2.44 and 1.92 Å, respectively (Figure S13, Supporting Information), whereas in **Sr–tbc** hybrids, these bond lengths were 2.07 and 1.83 Å (Figure 4g). These results suggested that **Sr–tbc** crystal possessed stronger C─H─O and O─H─O interactions compared to **Cd–tbc** crystal. The enhanced intermolecular interactions could facilitate the intermolecular charge transfer (CT) between **tbc** ^2−^ ligands, potentially leading to the multi‐peak emission of RTP.^[^ ^45^ ^]^

Furthermore, the face‐centered distances between two adjacent and parallel triazole rings in **Cd–tbc** and **Sr–tbc** were 4.91 and 3.82 Å, respectively (Figure 4h,i), meaning that significant π···π interactions were only present in **Sr–tbc**. Besides, the angles between two related axes of the triazole rings in **Cd–tbc** and **Sr–tbc** were 47.52° and 52.50°, respectively (*θ* < 54.7°), indicating the presence of *J*‐aggregation.^[^ ^46^ ^]^ Moreover, the face‐centered distance between two adjacent benzene rings was 3.80 Å in **Sr–tbc** crystals, which was shorter than that in **Cd–tbc** (4.43 Å). These results further suggested that **Sr–tbc** crystals exhibited the stronger π···π interactions. In conclusion, the intermolecular hydrogen bonds, coordination bonds, and π···π interactions could be tailored through coordination dimension engineering, collectively contributing to high‐efficiency RTP emission.

### 2.4 RTP Afterglow

**Figure 5:**
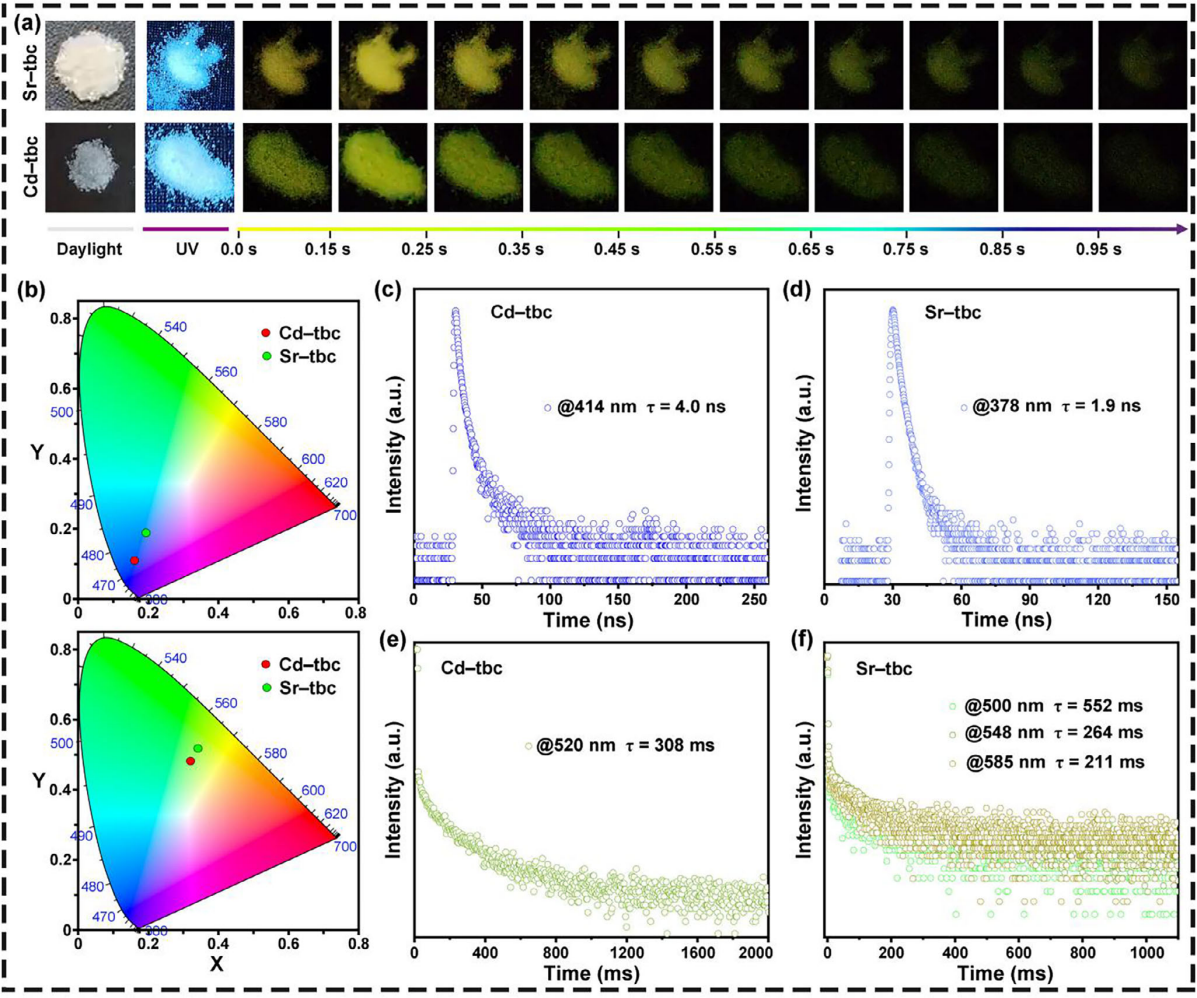
The afterglow properties of tbc‐based samples. a) Photographs of the RTP materials taken at different time intervals before and after turning off the UV excitation (365 nm); b) CIE system diagrams (top: fluorescence; down: phosphorescence); Time‐resolved c,d) fluorescence and e,f) phosphorescent decay curves at room temperature. All tests were conducted at room temperature, and the tested samples included the as‐prepared **Cd–tbc** and **Sr–tbc** crystals.

The afterglow properties of tbc‐based crystals were directly observed and recorded by optical photographs (**Figure** **5**a). Under daylight, both **Cd–tbc** and **Sr–tbc** crystals appeared as white powders. When exposed to UV irradiation at 365 nm, they exhibited a bright blue color. Upon cessation of UV excitation, the **Cd–tbc** MOFs displayed a noticeable green afterglow, which lasted for at last 0.7 s. Notably, the **Sr–tbc** MOF crystals demonstrated a unique color‐changing afterglow, transitioning from yellow to green over nearly 1.0 s. The long‐lasting, color‐shifting afterglow of the **Sr–tbc** crystals suggested significant potential for practical applications in areas, such as information confidentiality and optical recording devices.^[^ ^47^ ^]^

To understand the optical performances of these RTP crystals in depth, the steady‐state spectra of the synthesized **Cd–tbc** and **Sr–tbc** crystals were comprehensively investigated at room temperature. Both types of samples exhibited certain luminescence capabilities (Figure 5b,d). In comparison to the fluorescence excitation maximum of **Cd–tbc** crystals at 414 nm, **Sr–tbc** (378 nm) showed an obvious blueshift in overall emission wavelength (Figures S14 and S15, Supporting Information). The maximum phosphorescence emission position of the synthesized **Cd–tbc** crystal was 520 nm (Figure S14, Supporting Information). Notably, in addition to a main peak at 548 nm, the **Sr–tbc** crystal also displayed the two other distinct peaks at 500 and 585 nm in its maximum phosphorescence emission spectrum (Figure S15, Supporting Information). As observed in the CIE chromaticity diagrams, the corresponding color coordinates for the fluorescence emission of both types of crystals were concentrated in the blue‐purple region, while their corresponding color coordinates for the phosphorescence emission were concentrated in the yellow‐green region (Figure 5b). In essence, the diverse luminescence exhibited by these RTP crystals was attributed to their distinct molecular stacking arrangements.^[^ ^48^ ^]^

Although both the as‐synthesized **Cd–tbc** and **Sr–tbc** crystals exhibited the similar fluorescence lifetimes (less than 5.0 ns) (Figure 5c,d), they demonstrated the significantly diverse phosphorescence performances (Figure 5e,f). Compared to the as‐prepared 2D **Cd–tbc** (308 ms), the 1D **Sr–tbc** crystals displayed the longer RTP lifetimes of 552 ms. Notably, under the excitation of 360 nm, the **Sr–tbc** crystal exhibited the multimodal emission, with RTP lifetimes of 500, 548, and 585 nm at 552, 264, and 211 ms, respectively. Impressively, the **Sr–tbc** crystal achieved an RTP lifetime of 552 ms at 500 nm, surpassing the most metal–organic hybrid phosphors and qualifying as an ultralong RTP afterglow material (Tables S4 and S5, Supporting Information).^[^ ^49^, ^50^, ^51^, ^52^, ^53^, ^54^, ^55^, ^56^ ^]^ In addition, **Sr–tbc** (15.99%) demonstrated a higher photoluminescence quantum yield (PLQY) than **Cd–tbc** (4.70%), confirming its superior luminescent efficiency (Table S6, Supporting Information). Moreover, the luminescence efficiency of Sr‐tbc is inhibited by non‐radiative transitions, mainly due to C─H/N─H bond vibrations, residual water molecules, and crystal defects. The PLQY can be effectively enhanced by removing the solvent, isolating the matrix, and optimizing the crystal structure. These different RTP properties between the 2D **Cd–tbc** and 1D **Sr–tbc** crystals proved that the dimension engineering of metal–organic hybrids was a feasible strategy for the development of high‐efficiency RTP materials.

### 2.5 Optical Applications

**Figure 6:**
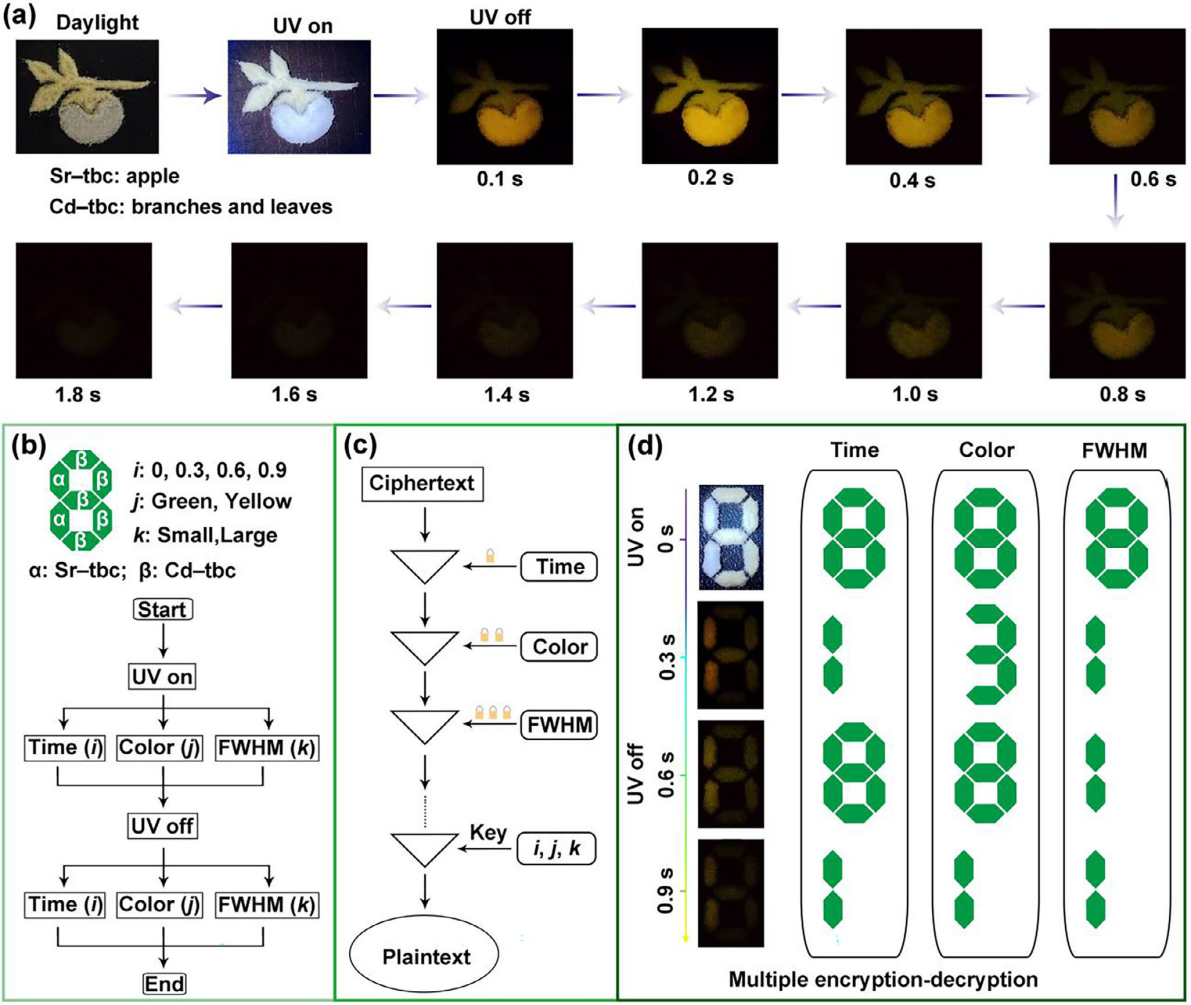
Multimodal encryption and dynamic afterglow display enabled by narrowband RTP materials. a) Time‐dependent afterglow decay patterns of a composite structure: **Sr–tbc** crystals (apple, from yellow to green emission) and **Cd–tbc** crystals (branches and leaves, green emission), illustrating programmable spatiotemporal resolution under UV cessation (λ_ex_ = 365 nm). b) The design of a multi‐channel encryption device integrating narrowband **Sr–tbc** and non‐narrowband **Cd–tbc** RTP materials. c) Hierarchical design diagram and d) process of multiple encryption–decryption with sequential for FWHM, color, and time‐resolved encryption under UV on and UV off.

To showcase the practical utility of **Sr–tbc** and **Cd–tbc**, their applications in high‐security data encryption and high‐quality dynamic afterglow displays were investigated (**Figure** **6**). A proof‐of concept composite model was fabricated by integrating **Sr–tbc** crystals (emulating an apple) and **Cd–tbc** crystals (simulating branches and leaves) (Figure 6a). Under ambient light, the composite exhibited a white apple against beige foliage. Upon UV excitation (λ_ex_ = 365 nm), the entire structure emitted the uniform blue luminescence. Critically, after UV cessation, time‐dependent afterglow behavior emerged. Specifically, branches/leaves (**Cd–tbc**) displayed green phosphorescence that decayed completely within 1.0 s. While apple (**Sr–tbc**) undergoes a dynamic color shift from luminous yellow to green, enabled by its narrowband RTP and multi‐emissive states. This spatiotemporally programmable afterglow highlighted the potential in dynamic optical encoding, where information was encrypted across spectral, chromatic, and temporal dimensions.

To achieve multi‐parameter anti‐counterfeiting, a hierarchical encryption platform was engineered by exploiting the orthogonal photophysical properties of **Sr–tbc** and **Cd–tbc** across temporal, chromatic, and spectral dimensions (Figure 6b–d). The encryption–decryption system capitalized on chromatic contrast (static green emission for **Cd–tbc**, time‐dependent yellow‐to‐green for **Sr–tbc**), temporal resolution (rapid decays for **Cd–tbc**, ultralong afterglow lifetime of 552 ms for **Sr–tbc**), and spectral distinction (non‐narrowband emission with FWHM of 120 nm for **Cd–tbc**, narrowband emission with FWHM of 28.5 nm for **Sr–tbc**) (Figure 6b,c). The encrypted information “8181,” “8381,” and “8111” was sequentially decrypted through three dimensions (Figure 6d). The first dimension was the prominent color along with the time dimension, and the sequence numbers were sequentially displayed as “8” for the fluorescence pattern, “1” for luminous yellow at 0.3 s, “8” for green at 0.6 s, and “1” for green at 0.9 s. The second dimension was green along with the color dimension, and the numbers were orderly displayed as “8” at 0 s, “3” at 0.3 s, “8” at 0.6 s, and “1” at 0.9 s. The third dimension was the FWHM values, and the numbers were orderly displayed as “8” for fluorescence emission at 0 s, “1” for narrowband RTP at 0.3, 0.6, and 0.9 s. This triple‐layered encryption strategy, integrating spectral fingerprinting, chromatic dynamics, and temporal gating, demonstrated the unparalleled utility of narrowband RTP materials in ultra–secure optical data storage and dynamic anti‐counterfeiting.

## 3 Conclusion

In conclusion, we have successfully developed **Sr–tbc**, the first non‐RE narrowband RTP MOF crystal, featuring 1D coordination chains via coordination dimension engineering. This material displayed distinct narrowband RTP emission across a broad excitation wavelength range (280–360 nm), with a minimum FWHM value of 28.5 nm. Moreover, when excited by light source between 260 and 300 nm, **Sr–tbc** crystals exhibited unusual high‐energy UV RTP emission at either 357 or 370 nm. Additionally, this crystal showed an ultralong phosphorescence lifetime of up to 552 ms (λ_em_ = 500 nm). These exceptional phosphorescence properties originated from a CLPA mechanism, which enhanced specific emission and rigidified the molecular arrangement via selective coordination interactions. This work established a novel CLPA strategy for designing high‐performance narrowband RTP materials and demonstrated their potential for advanced applications.

## Conflict of Interest

The authors declare no conflict of interest.

## Author Contributions

W.L. and J. L. contributed equally to this work. W.Z., Z.L., and C.L. conceived the experiments. W.Z. and J.L. prepared the paper. W.Z., M.Z., X.W., J.L., and T.L. were primarily responsible for the experiments and measurements. H.L. and M.W. contributed to calculations. M.Z., F.C., Z.L., and C.L. gave suggestions for the manuscript. All authors contributed to the data analyses.

## Supporting information



Supporting Information

## Data Availability

The data that support the findings of this study are available from the corresponding author upon reasonable request.
