# Supplementary material for: Coordination‐Locked Engineering to Achieve Narrowband Room Temperature Phosphorescence in Non‐Rare Earth Metal–Organic Frameworks
Source: Adv Sci (Weinh). 2025 Oct 22;13(3):e16309. doi: 10.1002/advs.202516309 (PMC12806520; doi:10.1002/advs.202516309)
Supplement: Supplementary file 1 — Supporting Information [file ADVS-13-e16309-s001.doc]

Supporting Information

Coordination-Locked Engineering to Achieve Narrowband Room Temperature Phosphorescence in Non-Rare Earth Metal–Organic Frameworks

Wenlei Zhang,^[a][+]^ Jinpeng Li,^[a][+]^ Mengyao Wang,^[a]^ Yining Zhao,^[a]^ Xi Zhang,^[a]^ Hongfeng Li,^[c]^ Jing Liu,^[a]^ Tianyang Li,^[a]^ Meng Zhang,^[a]^ Fei Chen,^[a]^ Zhongyi Liu,^[a]^* Chao Lu^[a][b]^*

^[a]^ College of Chemistry, State Key Laboratory of Coking Coal Resources Green Exploitation, Zhengzhou University, Zhengzhou 450001 (China)

^[b]^ State Key Laboratory of Chemical Resource Engineering, College of Chemistry, Beijing University of Chemical Technology, Beijing 100029 (China)

^[c]^ School of Intelligent Manufacturing, Huzhou College, Huzhou 313000 (China)

^*^Corresponding author. E-mail: liuzhongyi@zzu.edu.cn, luchao@mail.buct.edu.cn

^[+]^ These authors contributed equally to this work

Experimental Section

**Chemicals and Materials.** Strontium chloride (SrCl_2_, 99%, Macklin), cadmium sulfate hydrate (CdSO_4_·8/3H_2_O, 99%, Macklin), 1-(4-carboxyphenyl)-1H-1,2,4-triazole-3-carboxylic acid (H_2_tbc, AR, Jinan Henghua Technology Co., Ltd.), methanol (CH_3_OH, AR, Hushi Chemical Reagent Co., Ltd.), Acetonitrile (CH_3_CN, AR, Energy Chemical Reagent Co., Ltd.), are directly used without further purification.

**Synthesis of** **[Cd(tbc)_2_(H_2_O)_2_]_n_ (Cd−tbc)**. **Cd−tbc** bulk-shaped crystals were synthesized according to the procedure described in the previous literature.**^1^** The yield (80%, based on CdSO_4_·8/3H_2_O) was higher than that reported. Elemental Anal. Calcd. For C_20_H_18_Cd2N_6_O_12_: C, 31.64; H, 2.39; N, 11.07 (%). Found: C, 32.88; H, 2.88; N, 12.44 (%). IR (KBr pellet, cm^−1^): 3374w, 3119m, 2918w, 1605s, 1569s,1469s, 1296m, 1050w, 986m, 849m, 785m, 657w, 503w.

**Preparation of [Sr(Htbc)_2_(H_2_O)_3_]_n_ (Sr−tbc).** A mixture of SrCl_2_ (0.03 mmol, 4.8 mg) and H_2_tbc (0.03 mmol, 7.0 mg) was dissolved in a mixed solvent of H_2_O/CH_3_OH (2:3 by volume, 5 mL), stirred for 10 min to form a homogeneous solution, and then sealed in a 25 mL Teflon-lined stainless steel vessel. The mixture was slowly heated at 80 °C for 72 h and then cooled to room temperature at a rate of 10 °C·h^−1^. Colorless crystals of **Sr−tbc** were filtered, dried in the air at room temperature (yield: 78%, based on Sr). Elemental Anal. Calcd. For C_20_H_18_SrN_6_O_11_: C, 37.57; H, 2.71; N, 113.14 (%). Found: C, 36.99; H, 2.68; N, 12.44 (%). IR (KBr pellet, cm^−1^): 3389s, 3109m, 2922s, 2577s, 1918m, 1740w, 1659m, 1600m, 1521w, 1440m, 1319s, 1251s, 1079m, 981w, 890s, 810m, 765w, 689m, 650s, 573m, 455m.

**Apparatus and characterizations.**

All **elemental analysis** (C, H, and N) were performed on a FLASH EA 1112 instrument. Powder X-ray diffraction (PXRD) data were measured on a Bruker D8 VENTURE diffractometer with Cu-Kα radiation (λ = 1.5406 Å). The steady-state spectra (fluorescence and phosphorescence) for the solid samples were recorded on a HITACHI F-4600 fluorescence spectrophotometer at room temperature. The phosphorescence lifetimes were measured by a FLS980 fluorescence spectrometer.

**Crystal Data Collection and Refinement**. Single crystal of Cd-binc and Zn-binc with high quality were selected. Their X-ray diffraction data were collected on a Bruker SMART APEX-Ⅱ CCD diffractometer1 employing Mo-Kα radiation (λ = 0.71073 Å) at 298 ± 1 K. The structures of two crystals were solved with the SHELXS-97 crystallographic software package and refined through the SHELXL-2014 programs. All nonhydrogen atoms were refined anisotropically.

**Safety statements.** No unexpected or unusually high safety hazards were encountered.


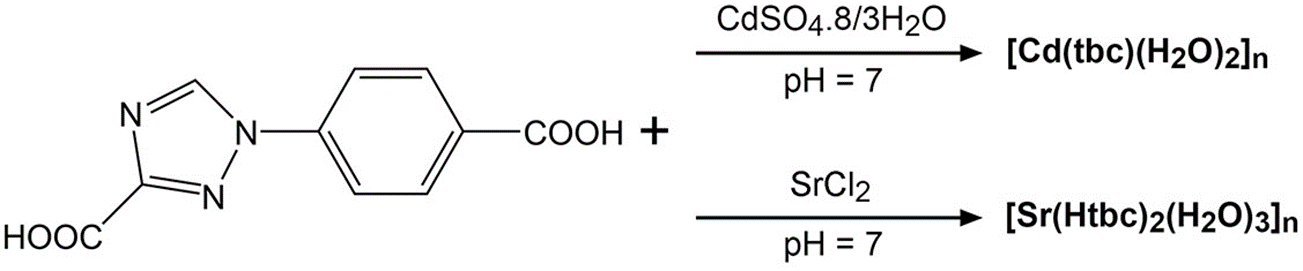


**Figure S1**. The schematic synthesized process of tbc-based crystalline frameworks, including **Cd−tbc** and **Sr−tbc**.


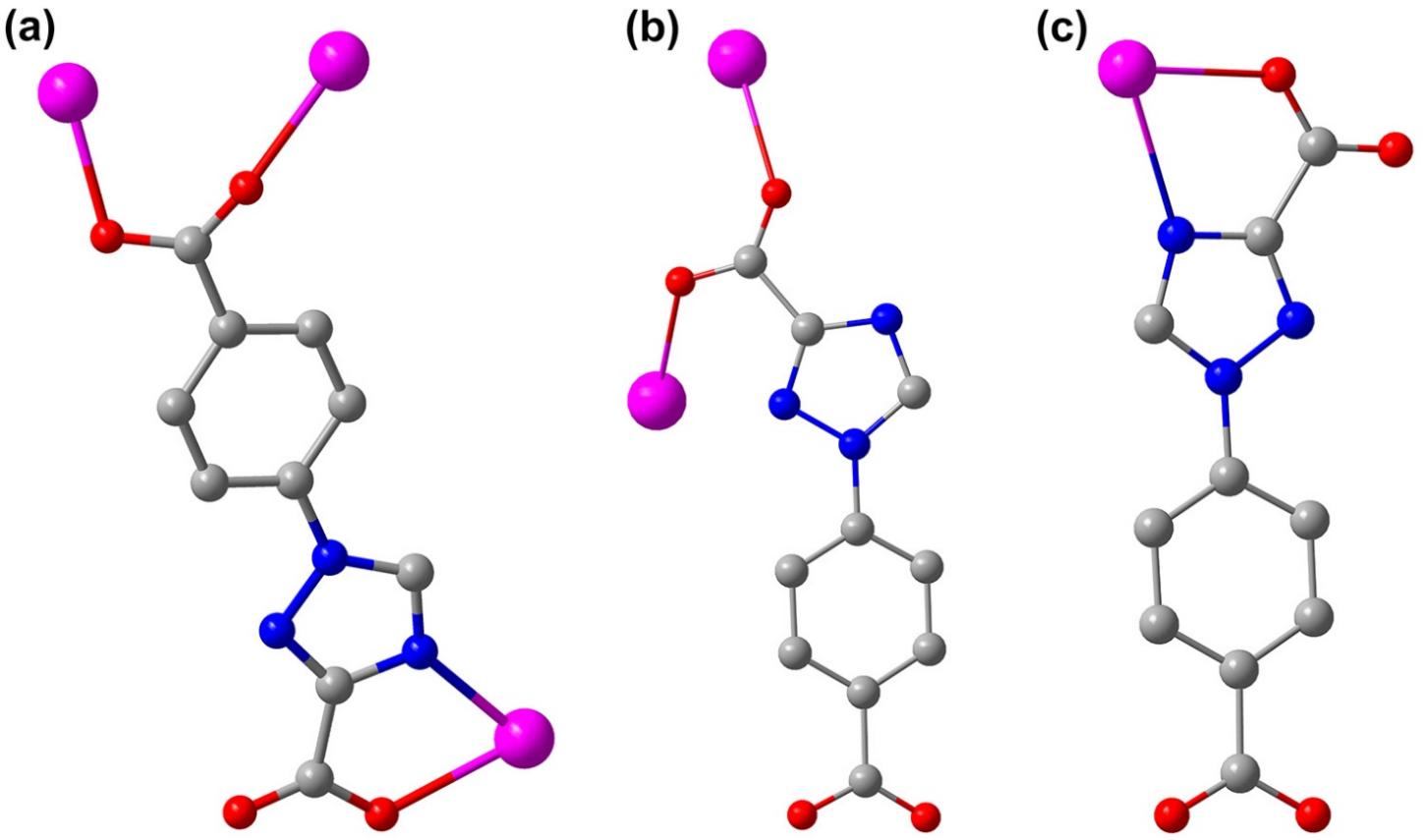


**Figure S2.** The coordination modes of **tbc^2−^** liands in compounds: (**a**) **Cd−tbc** and (**b-c**) **Sr−tbc**.

In **Sr−tbc** crystal, there are two kinds of coordination modes for the Htbc− ligand. Two oxygen atoms on a carboxyl group of Htbc^−^ ligand respectively coordinated with different Sr(II) ions (**Scheme S2b**), or the triazolium nitrogen atom and carboxyl oxygen atom of an Htbc^−^ ligand coordinated with one Sr(II) ions (**Scheme S2c**).


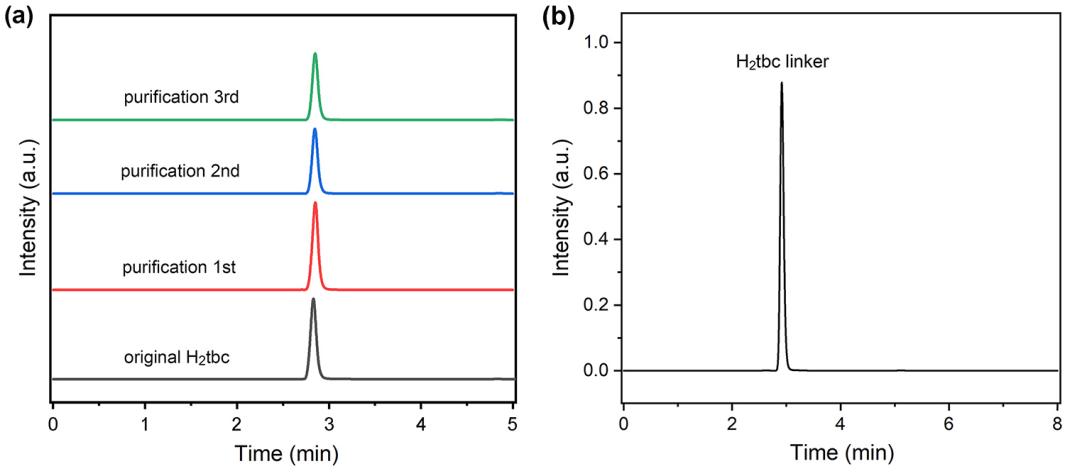


**Figure S3.** The high-performance liquid chromatography (HPLC) data of original and purified H_2_tbc solid.

..


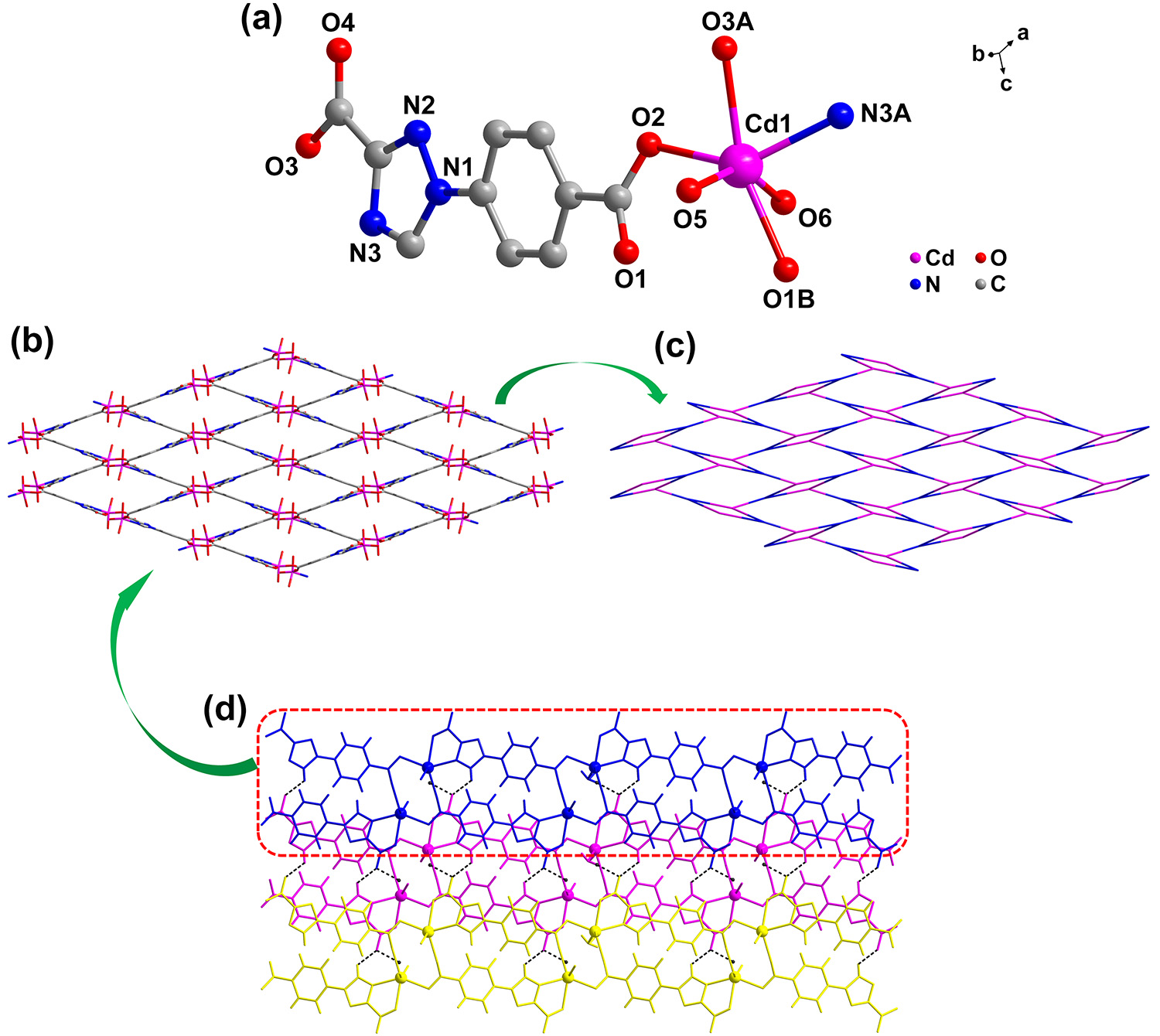


**Figure S4**. The assembly structure of **Cd−tbc**. (**a**) The coordination environment around the Cd(II) atom in **Cd−tbc**, all hydrogen atoms are omitted for clarity. (**b**) 2D net structure and (**c**) 2D topology diagram of **Cd−tbc**. (**d**) View of the 3D packing structure. Intermolecular interactions are denoted by dotted lines.

Single-crystal X-ray diffraction reveals that **Cd−tbc** crystallizes in the monoclinic system with the P*2*_1_/*c* space group. It should be noted that the structure of **Cd−tbc** has been reported, and the specific description of its crystal structure is omitted.**^1^** Herein, we only point out the spatial arrangement and intermolecular interactions in **Cd−tbc**. In the crystal, one tbc^2−^ ligand coordinate with three Cd(Ⅱ) ions (**Figure S4a**). In this coordination mode, two oxygen atoms (O1, O2) on one carboxyl group of the tbc^2−^ ligand respectively coordinate with one Cd(Ⅱ) ion, and the third Cd(Ⅱ) ion is chelated to one oxygen atom (O3) of another carboxyl group and the pyrazole nitrogen atom (N3). In **Cd−tbc**, adjacent Cd(II) ions are bridged by **tbc^−^** ligands to form an infinite one-dimensional chain (**Figure S4b-c**). These 1D chains are stacked into extended 2D network structure through monodentate coordination between carboxyl oxygen atoms and Cd(Ⅱ) ions. The two-dimensional structures are stacked into 3D supramolecular structure via intermolecular hydrogen bonds C3−H3···O4 [H/O distances (bond angles): 2.44 Å (123.20°)] and O8−H8···O4 [H/O distances (bond angles): 1.92 Å (153.58°)] (**Figure S4d**).


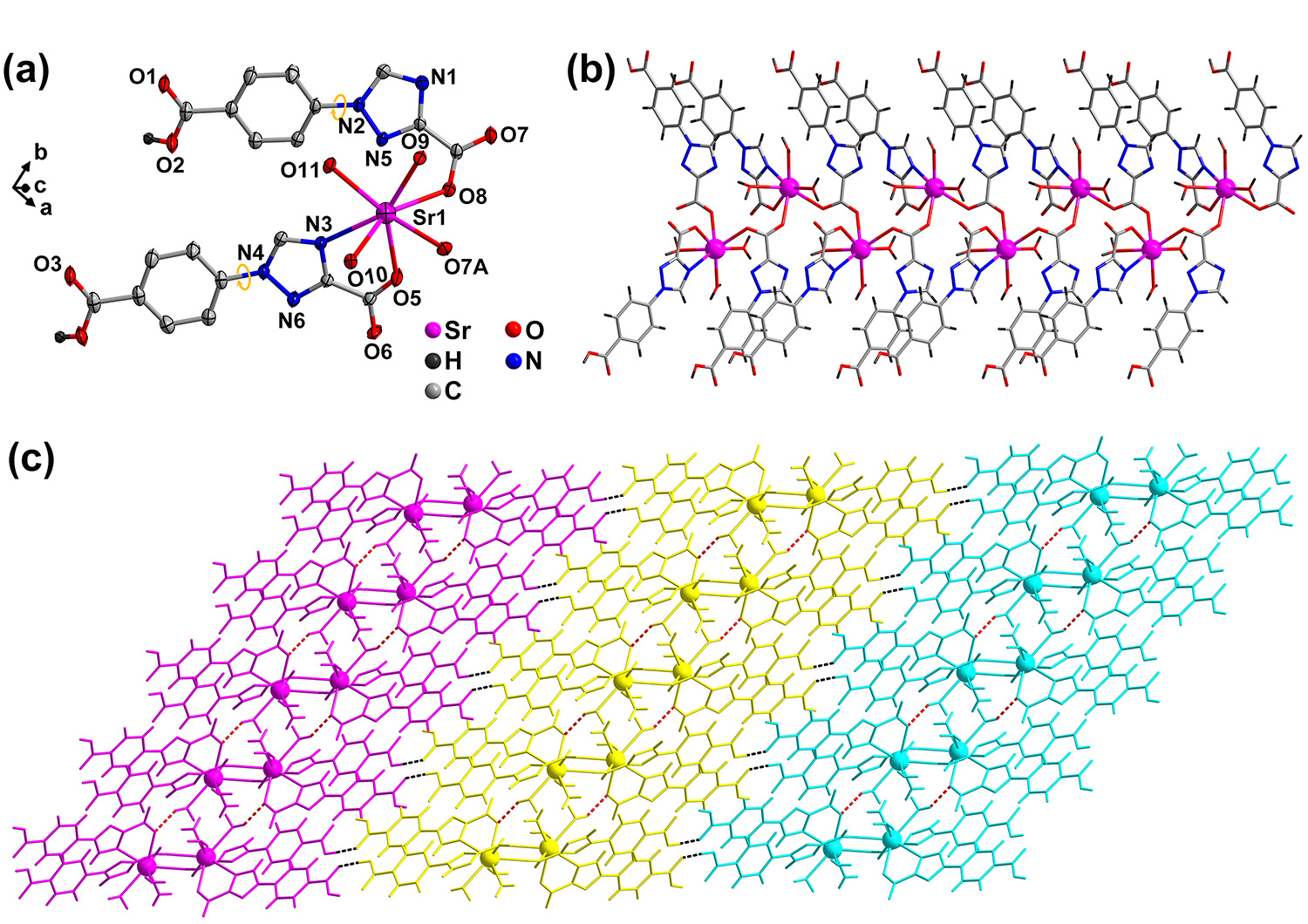


**Figure S5**. The assembly structure of **Sr−tbc**. (a) The surrounding coordination environment of **Sr−tbc**, all hydrogen atoms are omitted for clarity. (b) 1D chain diagram and (c) 3D stacking diagram. Intermolecular hydrogen bonds in **Sr−tbc** are denoted by dotted lines.

Single crystal X-ray diffraction analysis reveals that **Sr−tbc** is a mononuclear complex which crystallizes in the monoclinic system with space group *P2(1)/c*. There is one crystallographically independent Sr(II) ion, two H**tbc^−^** ligands and three coordinated water molecule in the asymmetric unit. As shown in **Figure S5a**, each seven-coordinated Sr(II) ion is coordinated to one triazolium nitrogen atoms (N3), three carboxyl oxygen atoms (O5, O7A, O8) from three different monodentate **Htcb^−^** ligands, and three oxygen atoms (O9, O10, O11) from three coordination water molecules. The Sr−O bond lengths vary from 2.55 to 2.61 Å, the Sr−N bond length is equal to 2.71 Å, and the angles around Sr(II) are in the range of 62.11º to 148.44º, which are in good accordance with the reported Sr(II) complexes.**^2-3^** 1D chain structure is formed through the monodentate coordination between two adjacent Sr(Ⅱ) ions and two carboxyl oxygen atoms of same Htbc^−^ ligand (**Figure S5b**). The 3D structure of **Sr−tbc** is further stabilized by the intermolecular hydrogen bonds, including C11−H11A···O5 (H11A···O5 = 2.07Å, C11−H11A···O5 = 149.1º) and O2−H2···O3 (H2···O3 = 1.83Å, O2−H2···O3 = 154.5º) (**Figure S5c**)..


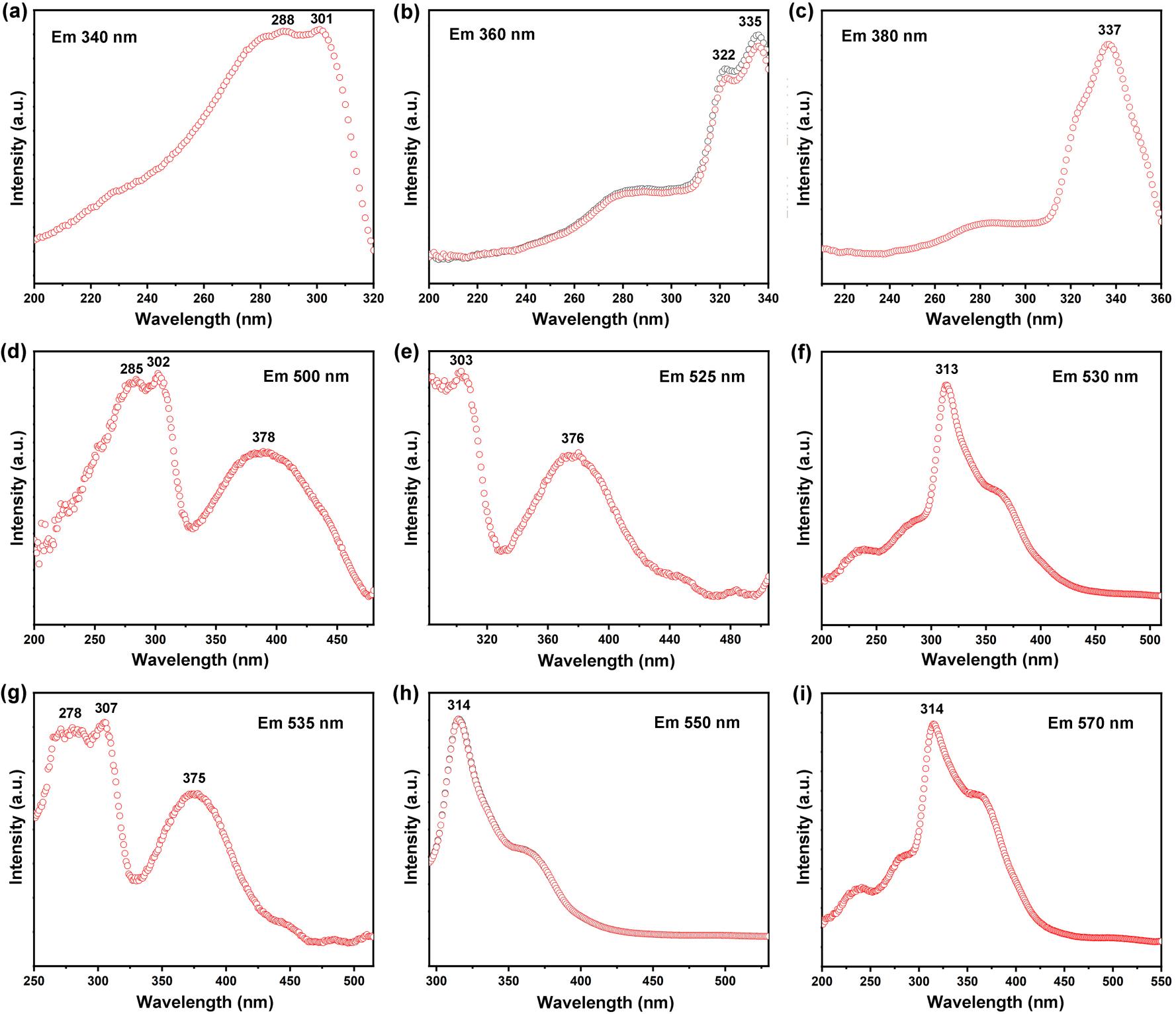


**Figure S6.** The room-temperature phosphorescence (RTP) spectra of **Sr–tbc** under fixed detection wavelengths (Em).


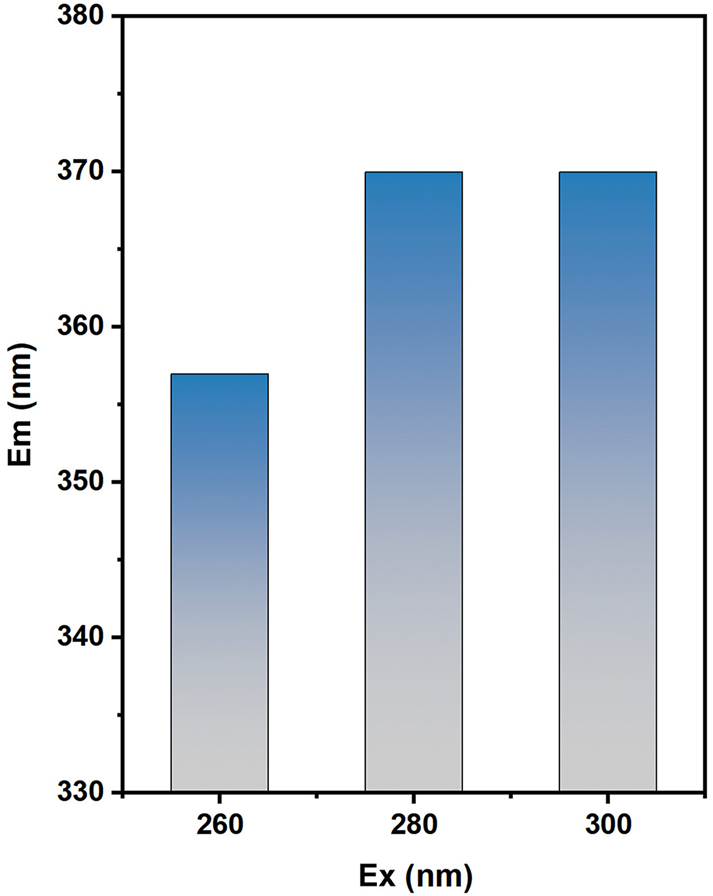


**Figure S7.** The ultraviolet RTP emission of **Sr–tbc**.


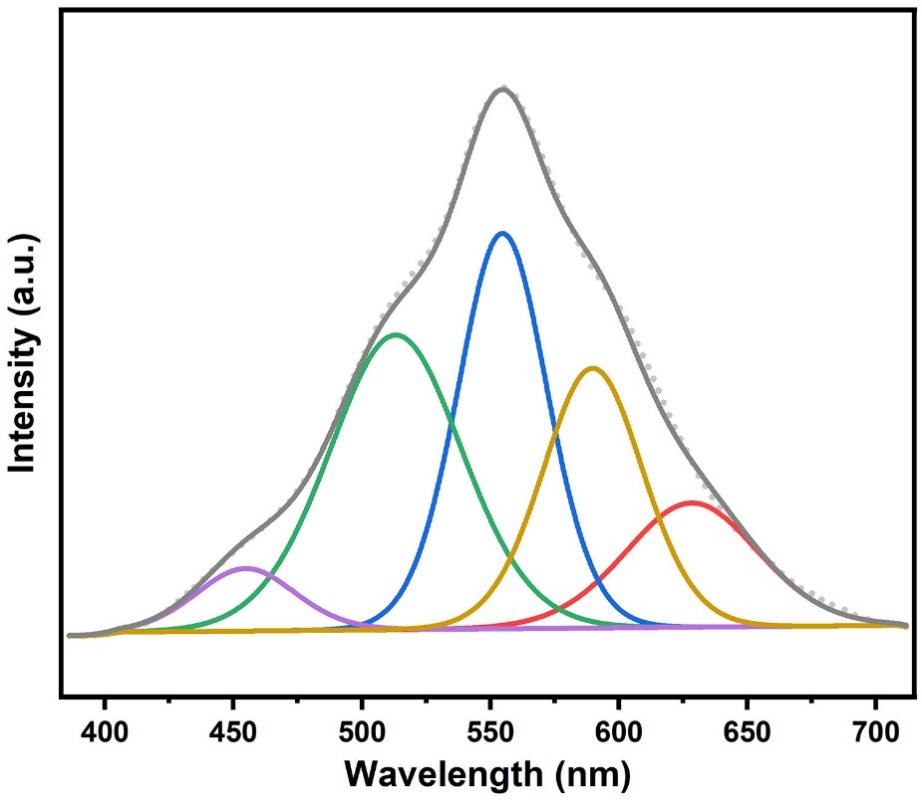


**Figure S8.** Deconvoluted phosphorescence emission bands of H_2_tbc.


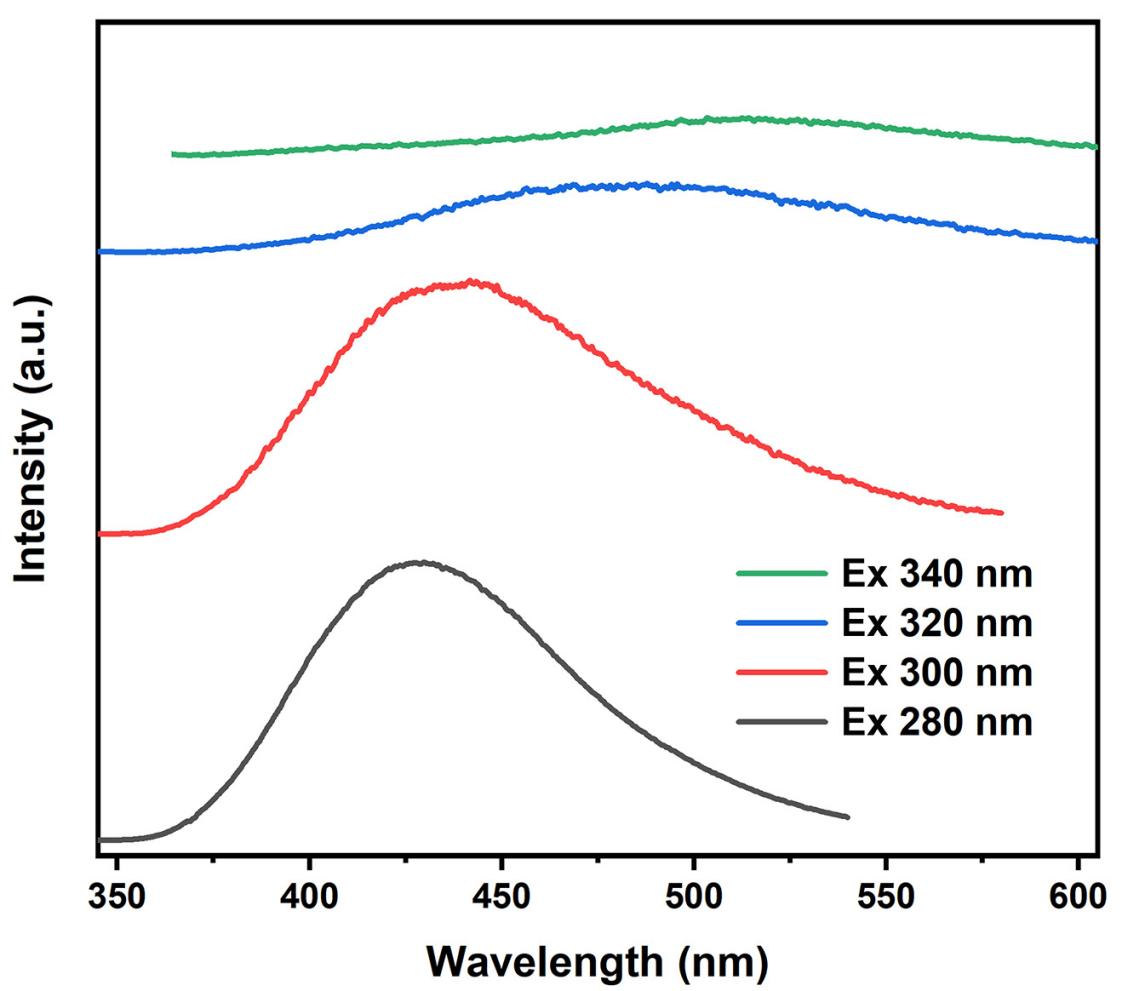


**Figure S9**. The RTP spectra of triazolinic acid (**TA**) under fixed excitation wavelengths (E_X_).


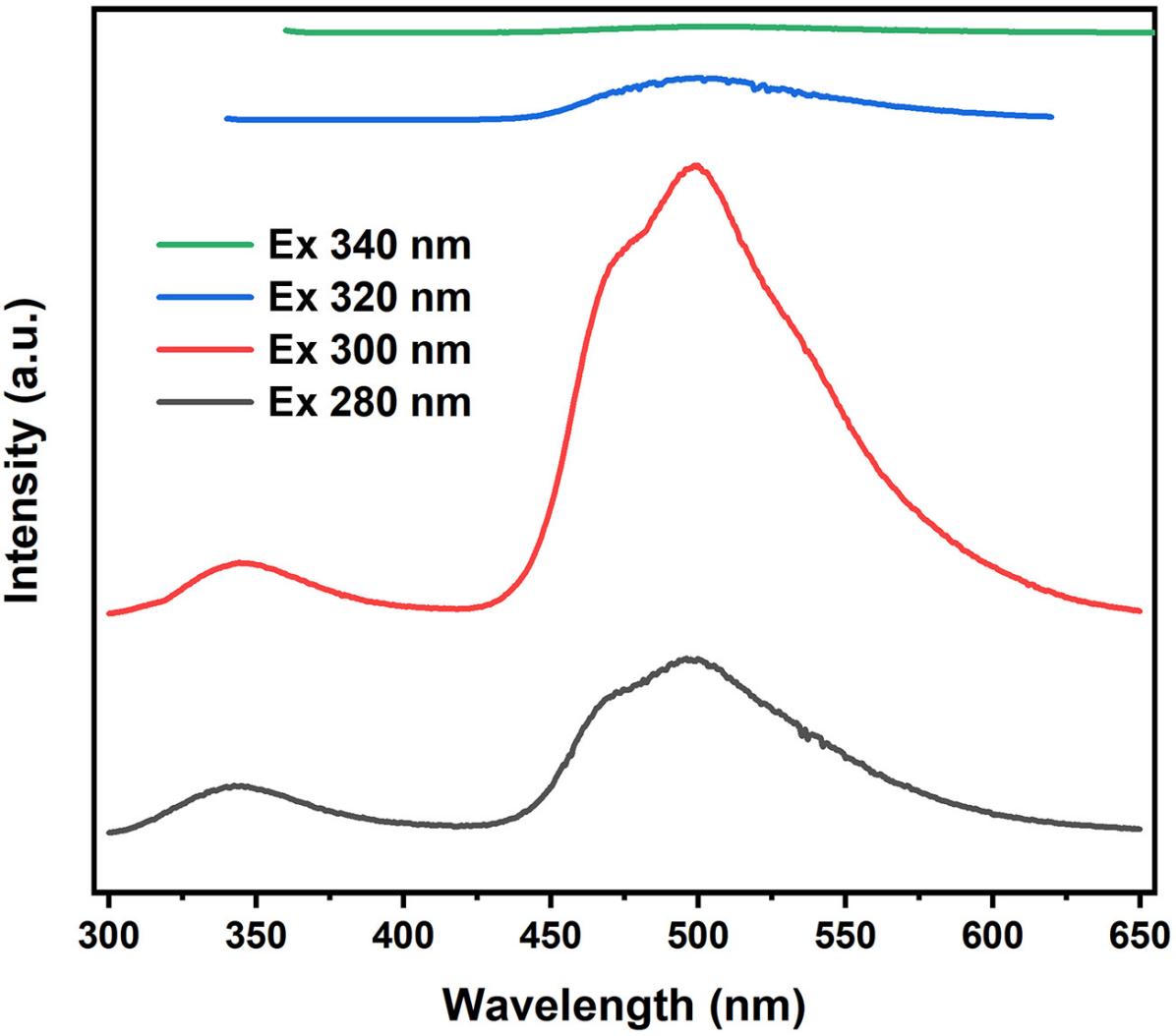


**Figure S10**. The RTP spectra of benzoic acid (**BA**) under fixed excitation wavelengths (E_X_).


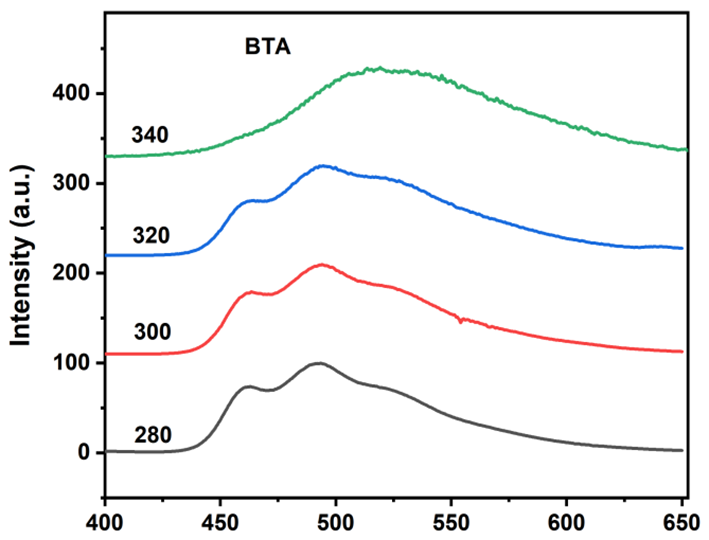


**Figure S11.** The RTP spectra of 1-(phenyl)-1H-1,2,4-triazole-3-carboxylic acid (**BTA**) under fixed excitation wavelengths (E_X_).


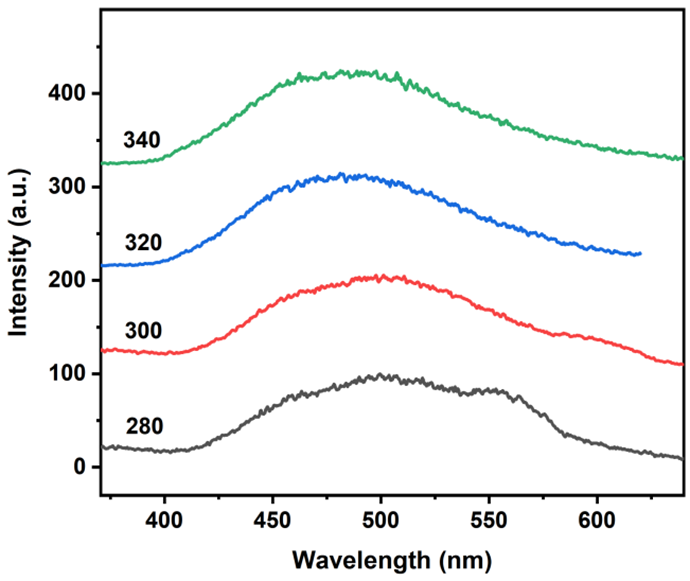


**Figure S12**. The RTP spectra of 1H-1,2,4-triazole-1-benzoic acid (**TBA**) under fixed excitation wavelengths (E_X_).


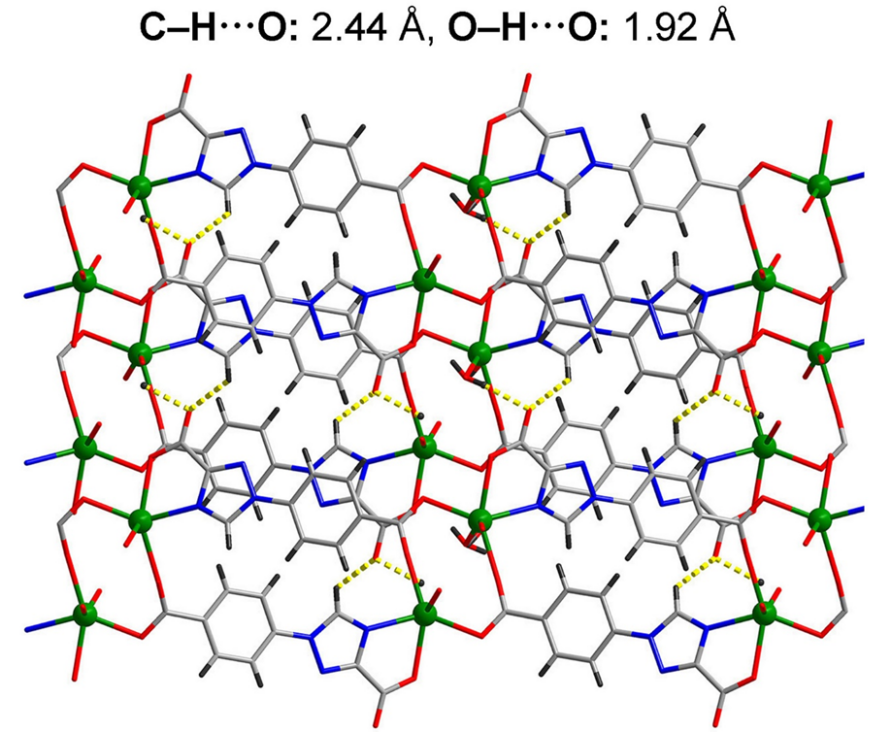


**Figure S13**. The stacking diagrams of three-dimensional **Cd–tbc** (intermolecular hydrogen bonds are indicated by dotted lines).


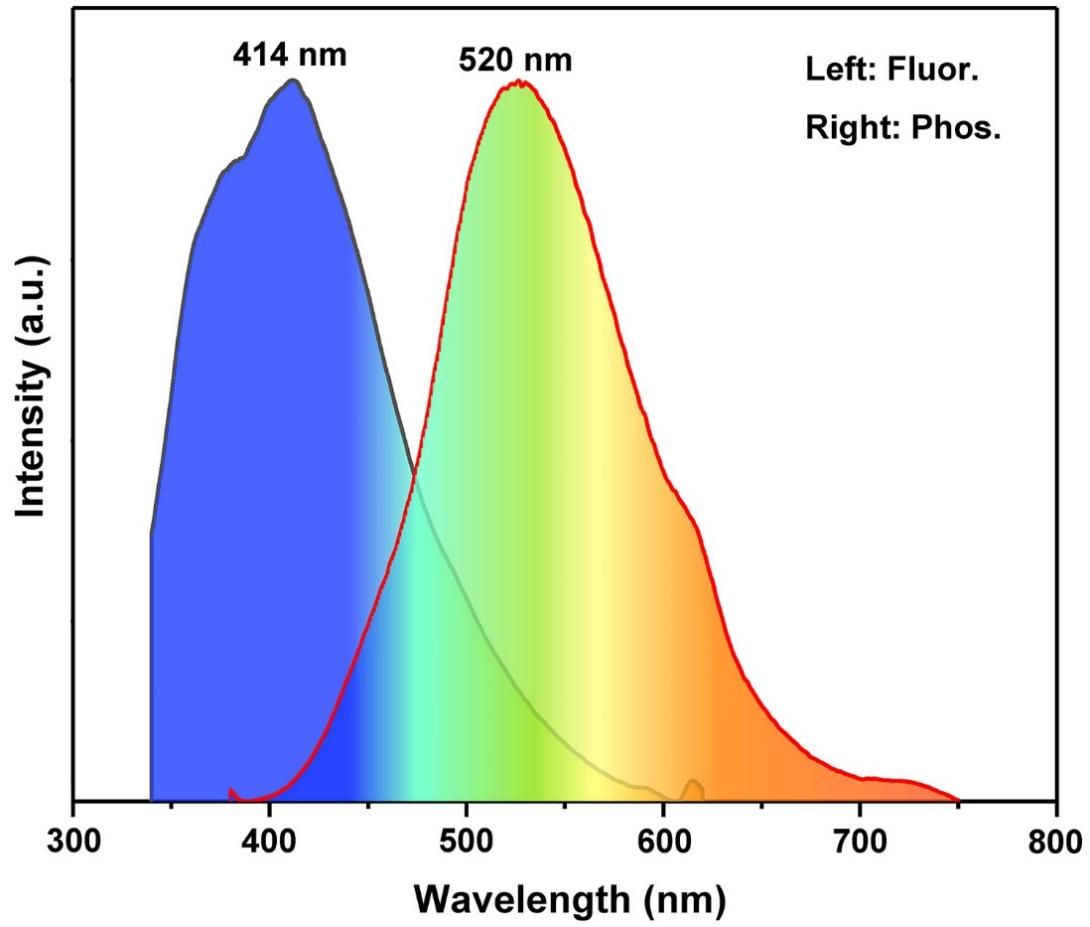


**Figure S14**. The normalized photoluminescence spectra of **Cd–tbc** at room temperature (the fluorescence spectrum curve is on the left side of the spectrum, and the phosphorescence spectrum curve is on the right side).


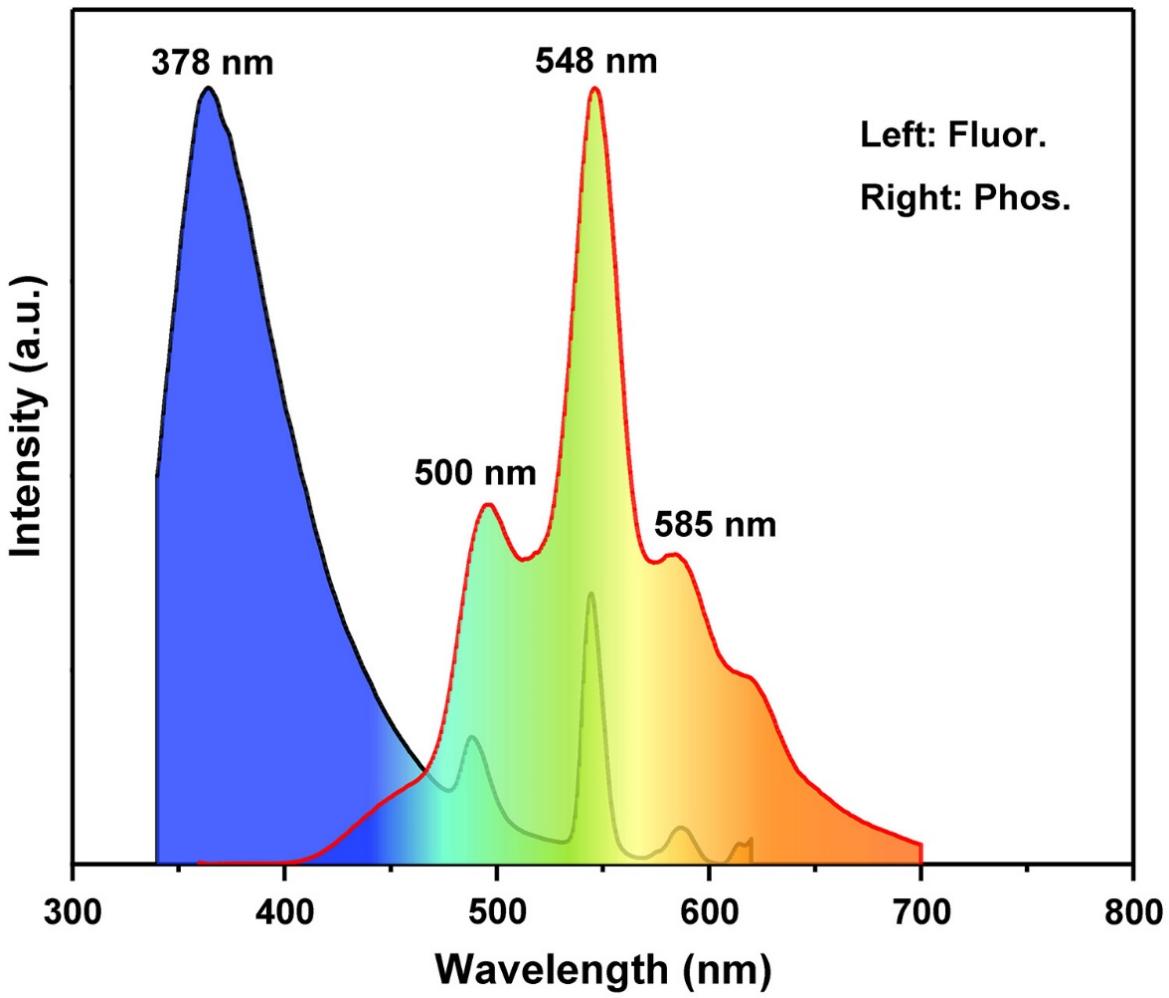


**Figure S15**. The normalized photoluminescence spectra of **Sr–tbc** at room temperature (the fluorescence spectrum curve is on the left side of the spectrum, and the phosphorescence spectrum curve is on the right side).


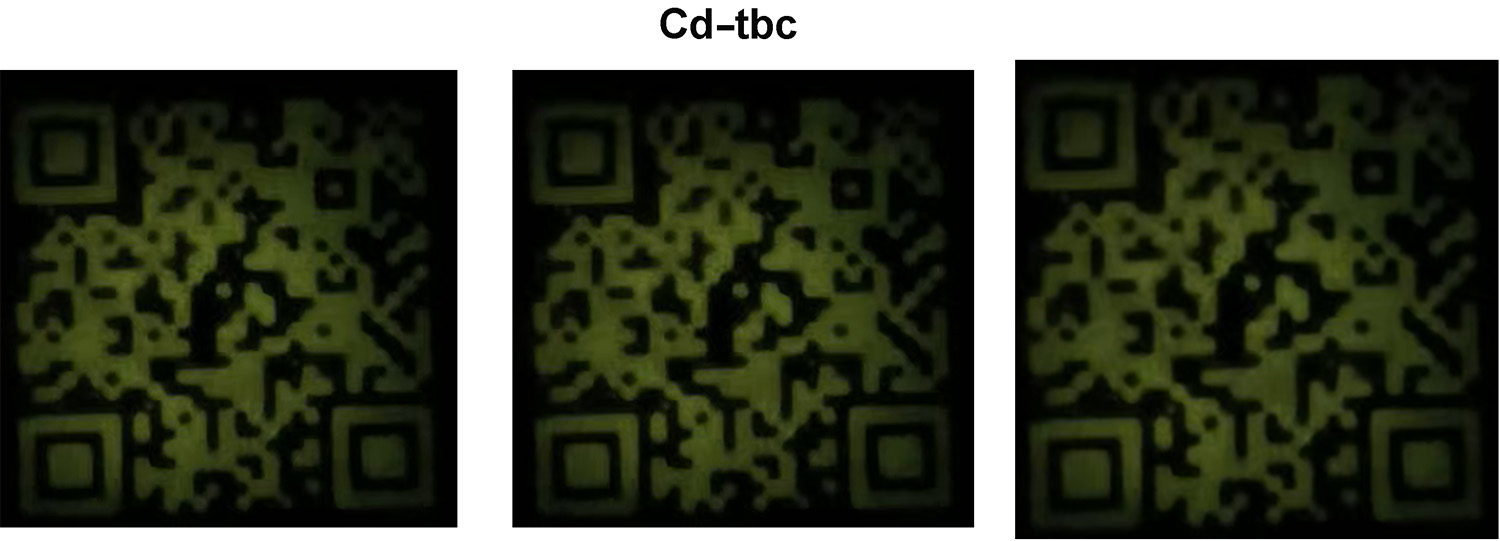


**Figure S16**. The anti-counterfeiting QR codes were fabricated using the RTP afterglow of the Cd-tbc crystal.


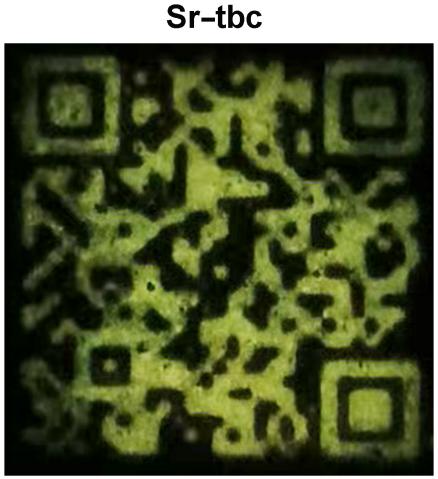


**Figure S17**. The anti-counterfeiting QR codes were fabricated using the RTP afterglow of the Sr-tbc crystal.

**Table S1.** The processing parameters and crystallographic data for **Cd−tbc**, and **Sr−tbc**.

| Compound | **Cd−tbc** | **Sr−tbc** |
| --- | --- | --- |
| Formula | C_20_H_18_Cd_2_N_6_O_12_ | C_20_H_18_SrN_6_O_11_ |
| Formula weight | 759.20 | 606.02 |
| Temperature (K) | 273(2) | 293(2) |
| Wavelength (Å) | 0.71073 | 0.71073 |
| Crystal system | Monoclinic | Monoclinic |
| Space group | *P2*_1_*/c* | *P2*_1_*/c* |
| *a* (Å) | 13.8220(19) | 7.2046(2) |
| *b* (Å) | 7.7638(11) | 9.0502(2) |
| *c* (Å) | 12.1900(16) | 34.9714(9) |
| *α* (deg) | 90 | 90 |
| *β* (deg) | 116.16 | 91.6380(10) |
| *γ* (deg) | 90 | 90 |
| V (Å^3^) | 1174.1(3) | 2279.31(10) |
| Z | 2 | 4 |
| Dc (g·cm^−3^) | 2.148 | 1.766 |
| F(000) | 744 | 1224 |
| θ range for data collection (deg) | 3.095−27.664 | 2.828−27.101 |
| Reflections collected/unique | 28804/2719 | 34343/5023 |
| Data/restraints/params | 2719/0/181 | 5023/0/347 |
| Goodness-of-fit on F^2^ | 1.001 | 1.085 |
| Final *R_1_*^a^, *wR_2_*^b^ | 0.0343, 0.0902 | 0.0286, 0.0674 |

*^a^R_1_ = ||F_o_| - |F_c_||/|F_o_|. ^b^wR_2_ =* [*w*(|*F_o_^2^| - |F_c_^2^|*)*2/w|F_o_^2^|^2^*]^1/2^*.*

**Table S2.** Selected bond lengths (Å) and angles (deg) for **Cd−tbc** and **Sr−tbc**.

| **Cd−tbc** |  |  |  |  |  |
| --- | --- | --- | --- | --- | --- |
| Cd(1)−O(2) | 2.234(2) | Cd(01)−N(3)#3 | 2.290(2) | O(5) −Cd(1)−O(1)#1 | 77.07(8) |
| Cd(1)−O(5) | 2.237(2) | O(2) −Cd(1)−O(5) | 97.78(8) | N(3)#1−Cd(1)−O(1)#2 | 89.52(8) |
| Cd(1)−N(3)#1 | 2.290(2) | O(2)−Cd(1)−N(3)#1 | 139.33(8) | O(6)−Cd(1)−O(1)#2 | 77.77(7) |
| Cd(1)−O(6) | 2.322(2) | O(5)−Cd(1)−N(3)#1 | 106.75(9) | O(2)−Cd(1)−O(3)#1 | 78.63(8) |
| Cd(1)−O(1)#2 | 2.329(2) | O(2)−Cd(1)−O(6) | 83.08(8) | O(5)−Cd(1)−O(3)#1 | 90.23(8) |
| Cd(1)−O(3)#1 | 2.396(2) | O(5)−Cd(1)−O(6) | 148.46(7) | N(3)#1−Cd(1)−O(3)#1 | 69.52(7) |
| O(3)−Cd(1)#3 | 2.396(2) | N(3)#1−Cd(1)−N(6) | 91.71(8) | O(6)−Cd(1)−O(3)#1 | 120.55(8) |
| O(1)−Cd(1)#2 | 2.329(2) | O(2)−Cd(1)−O(1)#2 | 127.97(8) | O(1)#2−Cd(1)−O(3)#1 | 151.41(8) |
| **Sr−tbc** |  |  |  |  |  |
| Sr(1)-O(8) | 2.5512(15) | O(9)-Sr(1)-O(5) | 146.16(5) | O(9)-Sr(1)-N(3) | 151.34(5) |
| Sr(1)-O(9) | 2.5608(16) | O(7)#1-Sr(1)-O(5) | 78.61(6) | O(7)#1-Sr(1)-N(3) | 131.85(5) |
| Sr(1)-O(7)#1 | 2.5691(15) | O(8)-Sr(1)-O(10) | 151.28(5) | O(5)-Sr(1)-N(3) | 62.11(5) |
| Sr(1)-O(5) | 2.5735(16) | O(9)-Sr(1)-O(10) | 104.41(6) | O(10)-Sr(1)-N(3) | 76.14(5) |
| Sr(1)-O(10) | 2.5966(16) | O(7)#1-Sr(1)-O(10) | 74.43(5) | O(11)-Sr(1)-N(3) | 81.43(5) |
| Sr(1)-O(11) | 2.6076(16) | O(5)-Sr(1)-O(10) | 86.12(6) | O(8)-Sr(1)-N(5) | 59.93(5) |
| Sr(1)-N(3) | 2.7129(17) | O(8)-Sr(1)-O(11) | 125.61(5) | O(9)-Sr(1)-N(5) | 75.19(5) |
| Sr(1)-N(5) | 2.8991(17) | O(9)-Sr(1)-O(11) | 70.84(5) | O(7)#1-Sr(1)-N(5) | 132.74(5) |
| O(7)-Sr(1)#2 | 2.5690(15) | O(7)#1-Sr(1)-O(11) | 127.51(6) | O(5)-Sr(1)-N(5) | 112.09(6) |
| O(8)-Sr(1)-O(9) | 74.08(5) | O(5)-Sr(1)-O(11) | 142.93(5) | O(10)-Sr(1)-N(5) | 148.44(5) |
| O(8)-Sr(1)-O(7)#1 | 77.77(5) | O(10)-Sr(1)-O(11) | 78.40(5) | O(11)-Sr(1)-N(5) | 71.65(5) |
| O(9)-Sr(1)-O(7)#1 | 73.68(5) | O(8)-Sr(1)-N(3) | 119.18(5) | N(3)-Sr(1)-N(5) | 89.56(5) |
| O(8)-Sr(1)-O(5) | 81.45(5) |  |  |  |  |

Symmetry transformations used to generate equivalent atoms:

For **Cd−tbc**: #1: x+1, -y+1/2, z+1/2; #2 -x+2, -y+1, -z+2; #3 x-1, -y+1/2, z-1/2.

For **Sr−tbc**: #1: -x+2, y-1/2, -z+1/2; #2 -x+2, y+1/2, -z+1/2.

**Table S3.** Photophysical properties of original H_2_tbc solid, crystalline **Cd−tbc** and **Sr−tbc**.

| **Sample** | **Fluorescence** | | | | | |  | **Phosphorescence** | | | | | |
| --- | --- | --- | --- | --- | --- | --- | --- | --- | --- | --- | --- | --- | --- |
|  | ***λ_em_***  **(nm)** | **m** | ***τ*_i_**  **(ns)** | **A_i_**  **(%)** | **<*τ*>** | **χ^2^** |  | ***λ_em_***  **(nm)** | **m** | ***τ*_i_**  **(ms)** | **A_i_**  **(%)** | **<*τ*>**  **(ms)** | **χ^2^** |
| **Cd−tbc** | 414 | 2 | 9.45  1.78 | 28.68  71.32 | 4.0 | 0.92 |  | 520 | 2 | 456  103 | 58.11  41.89 | 308 | 1.16 |
| **Zn−tbc** | 378 | 2 | 4.42  1.31 | 17.49  82.51 | 1.9 | 1.16 |  | 500 | 2 | 1667  166 | 25.68  74.32 | 552 | 1.27 |
|  |  |  |  |  |  |  |  | 548 | 2 | 60  332 | 25.23  74.77 | 264 | 1.15 |
|  |  |  |  |  |  |  |  | 585 | 2 | 278  65 | 68.38  31.62 | 211 | 1.26 |

***λ_em_*** = emission maximum, **m** stands for the i-exponential fitting of the PL decay curve, ***τ*_i_** is the excited state lifetime, **A_i_** represents the ratio of ***τ*_i_**. The fitting goodness is manifested by the value of **χ^2^** which should lower than 1.300. In the i-exponential case, <***τ***> = A_1_*τ_1_* + A_2_*τ_2_* + … + A_i_*τ_i_*, A_1_ + A_2_ + … +A_i_ = 1. **<*τ*> =** mean lifetime.

**Table S4. Photophysical properties of representative non-rare earth MOF RTP systems, including efficiency, FWHM, and lifetimes.**

| **Crystals** | **FWHM / nm** | **Lifetime / ms** |  | **PLQY / %** |
| --- | --- | --- | --- | --- |
| Zn-DCI-glass | 143 | 46.82 (453 nm)  197.15 (536 nm) |  | 75% |
| Cd-DCI-glass | 126 | 630.15 (441 nm)  219.05 (530 nm) |  | 58.4% |
| CAU-10-H | 104 | 638 |  | 25.2% |
| CAU-10-CH_3_ | 122 | 101 |  | 7.7% |
| CAU-10-OCH_3_ | 141 | 6.5 |  | 4.4% |
| ZnIPA | 76 | 475 |  | 17.5% |
| ZnTPA | 98 | 1300 |  | 11.1% |
| TMA | 62 | 163.7 |  | 18.2% |
| TDMA | 67 | 186.2 |  | 40.0% |
| TDEA | 68 | 169.7 |  | 80.6% |
| Zn-DCPS-BIMB | 162 | 238.2 |  | 1.32% |
| ZnCl_2_(BPP) | 127 | 228.31 |  | -- |
| ZnBr_2_(BPP) | 141 | 26.1 |  | -- |
| CP-Br | 118 | 1.55 (410nm)  14.63 (530nm)  17.57 (560nm) |  | -- |
| CP-Cl | 142 | 3.02 (410nm)  23.18 (530nm)  10.79 (550nm) |  | -- |
| QDU-21a | 91 | 20 |  | 13.8% |
| QDU-21b | 132 | 18 |  | 15.1% |
| MOF-5 | 102 | 539 |  | 5.4% |
| MOF-a | 126 | 180 |  | 28.7% |
| MOF-b | 53 | 1.1 |  | 24.9% |
| ZnCa-H_2_O | 106 | 54.2 |  | 1.0% |
| ZnCa-DMA | 113 | 14.5 |  | 3.7% |
| ZnCa-DEF | 151 | 76.5 |  | 1.3% |
| ZnMg-DMF | 122 | 21.5 |  | 2.3% |
| ZnCa-DMF | 88 | 48.8 |  | 3.0% |
| ZnSr-H_2_O | 93 | 195.4 |  | 6.8% |
| ZnBa-H_2_O | 97 | 153.8 |  | 9.1% |
| **Cd−tbc** | 119 | 308 |  | 4.70% |
| **Sr−tbc** | 28.5 | 552 (500 nm) |  | **15.99%** |
|  |  | 264 (548 nm) |  |  |
|  |  | 211 (585 nm) |  |  |

**Table S5. Structural and photophysical properties of reported transition metal coordination polymers afterglow.** The emission peaks (*l)* and lifetimes (*τ*) of the fluorescence and phosphorescence of the coordination polymers afterglow in the solid state.

| Complexes | Formula | Fluorescence | | Phosphorescence | | | Ref. |
| --- | --- | --- | --- | --- | --- | --- | --- |
|  |  | *l* (nm) | *τ* (ns) | *l* (nm) | *τ*_1_ /*τ*_2_ (ms) | Single/Dual |  |
| **Zn-TPA** | C_8_H_8_ZnO_6_ | 360 | - | 508 | 480 | Single | 4 |
| **Zn-IPA** | C_16_H_8_Zn_2_O_8_ | 387 | - | 484 | 1320 | Single | 4 |
| **Zn-TMA** | C_27_H_21_Zn_3_O_26_ | 334 | - | 533 | 200 | Single | 4 |
| **Cd-TPA** | C_25_H_29_Cd_2_N_3_O_11_ | 340 | - | 497 | 160 | Single | 4 |
| **Cd-11** | C_11_H_11_CdNO_5_ | 363 | - | 489 | 320 | Single | 4 |
| **Ca-EBTC** | C_24_H_22_CaN_2_O_10_ | 369 | 8.53 | 570 | 0.001 | Single | 5 |
| **Sr-EBTC** | C_40_H_26_Sr_3_O_18_S_2_ | 387 | 2.55 | 567 | 0.56 | Single | 5 |
| **Ba-EBTC** | C_44_H_38_Ba_3_O_20_S_4_ | 406 | 1.06 | 555 | 1.596 | Single | 5 |
| **CP1** | C_8_H_6_CdO_5_ | 392 | - | 514 | 700 | Single | 6 |
| **CP2** | C_30_H_15_Cd_2_N_4_O_8_ | 396 | - | 514 | 760 | Single | 6 |
| **1-DMF** | C_11_H_6_ZnNO_5_ | 326 | - | 503 | 470 | Single | 7 |
| **MOF-5** | C_11_H_6_ZnNO_5_ | 434 | - | 513 | 150 | Single | 7 |
| **Zn-1** | C_33_H_29_Zn_3.5_N_3_O_19_S_4_ | 436/464 | - | 495 | 4.57 | Single | 8 |
| **Zn-2** | C_16_H_10_ZnN_2_O_4_S_2_ | 550 | - | 550 | 0.16 | Single | 8 |
| **Zn5** | C_36_H_24_Br_4_N_2_O_20_ | 485 | 3.0 | 512 | 7.04 | Single | 9 |
| **Zn3** | C_14.5_H_12_BrN_3_O_6_Zn_1.5_ | 445 | 2.4 | 500 | 3.15 | Single | 9 |
| **Zn-BTC** | C_18_H_28_O_23_Na_2_Zn_2_ | 450 | - | 455/482/525 | 92/97/351 | Three | 10 |
| **1** | C_16_H_22_CdN_8_O_10_ | 399 | 4.5 | 508 | 43 | Single | 11 |
| **2** | C_30_H_26_CdN_12_O_5_ | 419 | 5.2 | 508 | 129 | Single | 11 |
| **3** | C_16_H_12_CdN_8_O_5_ | 420 | 6.5 | 510 | 179 | Single | 11 |
| **4** | C_16_H_14_CdN_8_O_6_ | 417 | 4.6 | 512 | 201 | Single | 11 |
| **5** | C_16_H_10_CdN_8_O_4_ | 422 | 8.8 | 543 | 41/144 | Dual | 11 |
| **6** | C_34_H_26_CdN_12_O_4_ | 419 | 5.7 | 520 | 38/183 | Dual | 11 |
| **7** | C_38_H_28_CdN_12_O_4_ | 430 | 6.1 | 561 | 52/194 | Dual | 11 |
| **8** | C_32_H_32_CdN_8_O_7_ | 423 | 4.8 | 555 | 62/236 | Dual | 11 |
| **Cd−tbc** | C_20_H_18_Cd_2_N_6_O_12_ | 414 | 4.0 | 520 | 308 | Single | **This work** |
| **Sr−tbc** | C_20_H_18_SrN_6_O_11_ | 378 | 1.9 | 500/548/585 | 552/264/211 | Three | **This work** |

**Table S6.** Quantum yield of **tbc**-based MOFs.

| Sample | Scatter Range | Emission Range | Quantum Yield |
| --- | --- | --- | --- |
| **Cd−tbc** | 300.5-341.5 nm | 342.5-700.0 nm | 4.70% |
| **Sr−tbc** | 310.0-345.5 nm | 347.0-800.0 nm | **15.99%** |

**References**

[1] S.-S. Zhang, Y.-T. Yan, W.-Y. Zhang, Y.-K. Fan, Y. Zhang, K. Zhong, Y.-Y. Wang, *Inorg. Chim. Acta* **2019**, *495*, 118971.

[2] W. Chen, J. Wang, L. Zhao, W. Dai, Z. Li, G. Li, *J. Alloys Compd.* **2018**, *750*, 895‒901.

[3] M. Usman, P.-H. Feng, K.-R. Chiou, J.-W. Chen, L.-W. Lee,Y.-H. Liu, K.-L. Lu, *ACS Appl. Electron. Mater.* **2019**, *1*, 836‒844.

[4] X. Yang, D. Yan, *Adv. Opt. Mater.* **2016**, *4*, 897‒905.

[5] L. Zhai, Z.-X. Yang, W.-W. Zhang, J.-L. Zuo, X.-M. Ren, *J. Mater. Chem. C* **2018**, *6*, 7030‒7041.

[6] Y. Yang, K.-Z. Wang, D. Yan, *ACS Appl. Mater. Interfaces* **2016**, *8*, 15489‒15496.

[7] X., Yang, D. Yan, *Chem. Sci.* **2016**, *7*, 4519‒4526.

[8] X.-G. Yang, Z.-M. Zhai, X.-Y. Liu, J.-Y. Li, F.-F. Li, L.-F. Ma, D*alton. Trans.* **2020**, *49*, 598‒602.

[9] D. Li, X. Yang, D. Yan, *ACS Appl. Mater. Interfaces* **2018**, *10*, 34377‒34384.

[10] H. Zhang, Y. Yan, G. Qiao, J. Li, *Inorg. Chem. Commun.* **2019**, *104*, 119‒123.

[11] Y. Li, T. Gai, Y. Lin, W. Zhang, K. Li, Y. Liu, Y. Duan, B. Li, J. Ding, J. Li, *Inorg. Chem. Front.* **2020**, *7*, 777‒785.
